# TGF-β Promotes Endothelial-to-Mesenchymal Transition and Alters Corneal Endothelial Cell Migration in Fuchs Endothelial Corneal Dystrophy

**DOI:** 10.3390/ijms26146685

**Published:** 2025-07-11

**Authors:** Judy Yan, Brooke Lim, Narisa Dhupar, Kathrine Bhargava, Lina Chen, Greg Moloney, Stephan Ong Tone

**Affiliations:** 1 Sunnybrook Research Institute, Toronto, ON M4N 3M5, Canada; 2 Department of Laboratory Medicine and Pathobiology, University of Toronto, Toronto, ON M5S 3K3, Canada; 3 Kensington Eye Institute, Toronto, ON M5S 1A8, Canada; 4 Department of Ophthalmology and Vision Sciences, University of British Colombia, Vancouver, BC V5Z 3N9, Canada; 5 Department of Ophthalmology and Vision Sciences, University of Toronto, Toronto, ON M5T 3A9, Canada

**Keywords:** Fuchs, cornea endothelial cells, transforming growth factor-beta, migration, endothelial-to-mesenchymal transition

## Abstract

Fuchs endothelial corneal dystrophy (FECD) is a progressive corneal disease characterized by corneal endothelial cell (CEC) loss and guttae formation. Elevated levels of Transforming Growth Factor-Beta 1 and 2 (TGF-β1/-β2) have been reported in the aqueous humor (AH) of FECD patients and have been implicated with abnormal extracellular matrix (ECM) production, endothelial-to-mesenchymal transition (EndoMT), the unfolded protein response, and cell death. However, how TGF-β signaling affects cell migration in FECD remains to be elucidated. In this study, we found that TGF-β2 levels were significantly elevated in the AH of FECD patients compared to controls. We performed bulk RNA sequencing on FECD CECs treated with TGF-β1 or TGF-β2 and identified the epithelial-to-mesenchymal (EMT) pathway as one of the top dysregulated pathways. We found that TGF-β1 and TGF-β2 increased EMT markers, filamentous-actin (F-actin) expression and produced more EMT-like phenotype in FECD and control CECs. We also observed that TGF-β1 and TGF-β2 significantly increased FECD CEC migration speed as detected by scratch assay and individual cell tracking and promoted individual cellular migration behavior. This study provides novel insight into FECD pathogenesis and how increased TGF-β signaling promotes EndoMT and alters cellular migration in FECD CECs.

## 1. Introduction

Fuchs endothelial corneal dystrophy (FECD) is a progressive, bilateral corneal endothelial disease that primarily occurs in women and leads to corneal dysfunction and vision loss. It typically presents in individuals in their fourth or fifth decade of life, affects 4–20% of people over the age of 40, and is the primary reason for corneal transplantation worldwide [1,2]. FECD pathogenesis is complex, with both genetic and environmental influences contributing to its onset [2,3]. Pathological characteristics of FECD include the loss of corneal endothelial cells (CECs), which disrupts corneal function, the accumulation of extracellular matrix (ECM) components, irregular Descemet’s membrane (DM) thickening, and the formation of guttae—an excess of collagen on DM [4,5]. FECD disease progression can lead to clinical manifestations of corneal edema, blurred vision, and blindness [6].

The corneal endothelium (CE) is composed of hexagonal corneal endothelial cells (CECs) arranged as a single monolayer on the posterior side of the cornea [7]. Its primary role is to maintain corneal deturgescence and transparency by actively regulating fluid movement between the stroma and the aqueous humor [8]. Corneal endothelial cell density gradually decreases with age, with cell density averaging 2600–3000 cells/mm^2^ in healthy adults [9]. When cell density falls below a critical threshold, the function of the CE becomes compromised resulting in inadequate fluid regulation, decreased clarity and corneal edema [9]. Pathological conditions, including FECD can accelerate cell loss, further compromising the corneal endothelial function. CECs are quiescent with limited proliferative capacity and remain arrested in the G1 phase of the cell cycle [2,4]. Consequently, with the loss of CECs in FECD, the CE relies heavily on cell migration and cell enlargement to facilitate wound healing [4,10,11,12]. Previous studies have investigated CEC migration in normal CE [13,14,15,16] and immortalized cells [17,18,19,20,21]. We have previously demonstrated that FECD CECs display increased cellular migration speeds compared to normal controls as observed in FECD cell lines and ex vivo patient specimens [22,23]. Epithelial-to-mesenchymal transition (EMT) is characterized by the shift of epithelial cells into a mesenchymal phenotype, which can result in increased cellular migration [24]. In the CE, CECs can also undergo EMT, a process referred to as endothelial-to-mesenchymal transition (EndoMT). Previous studies have reported that Transforming Growth Factor-Beta (TGF-β) is a key inducer and regulator of EndoMT under biological and pathological conditions [25,26].

TGF-β is a multifunctional cytokine involved in a range of cellular functions, including proliferation, differentiation, extracellular matrix (ECM) production, migration, EndoMT and EMT [27]. With three isoforms of TGF-β (−1, −2, −3) identified in mammals [28,29], TGF-β2 is reported to be the predominant isoform found in the aqueous humor (AH) [30]. In wounded corneas, TGF-β1 and TGF-β2, but not TGF-β3, were detectable in the anterior segment of the eye [28]. TGF-β has been shown to maintain corneal transparency by regulating ECM balance, preventing excessive deposition of collagen and other ECM components [28]. In addition, TGF-β promotes corneal wound healing by stimulating cell migration, proliferation, and tissue repair [31]. In CECs, TGF-β has been reported to reinforce cell junctions, contributing to the formation of a functional endothelial monolayer [32]. However, in FECD, aberrant TGF-β levels have been reported, suggesting a role in disease pathogenesis. Elevated levels of TGF-β in the CE have been reported to induce cell death via the unfolded protein response (UPR) [33]. Furthermore, inhibition of TGF-β represses excessive ECM formation in FECD [34]. While previous studies have reported that excessive TGF-β induces fibrotic changes in CECs, with increased ECM deposition and subsequent endothelial dysfunction [35], the effects of TGF-β on CEC migration in FECD are not well understood. This knowledge is especially pertinent to current treatments such as Descemet’s Stripping Only (DSO), a surgical technique that relies on peripheral CEC migration to regenerate the CE after removing the diseased central CE in FECD patients [36,37,38,39,40]. Given the crucial role of cell migration in corneal repair and maintenance, understanding how TGF-β influences this process will provide valuable insight into FECD pathogenesis.

In this study, we measured TGF-β levels in the AH of FECD patients and cataract control patients undergoing intraocular surgery. To investigate TGF-β-mediated effects in FECD, we performed bulk RNA sequencing on FECD CECs treated with TGF-β1 or TGF-β2. We explored whether TGF-β1 and TGF-β2 (1) increased EMT markers, (2) altered the morphology and expression of cytoskeleton components such as filamentous-actin (F-actin), and (3) increased cellular migration speeds and altered behavior of FECD CECs and normal controls.

## 2. Results

### 2.1. Elevated Levels of TGF-β2 in Aqueous Humor of Fuchs Endothelial Corneal Dystrophy Patients

**Figure 1:**
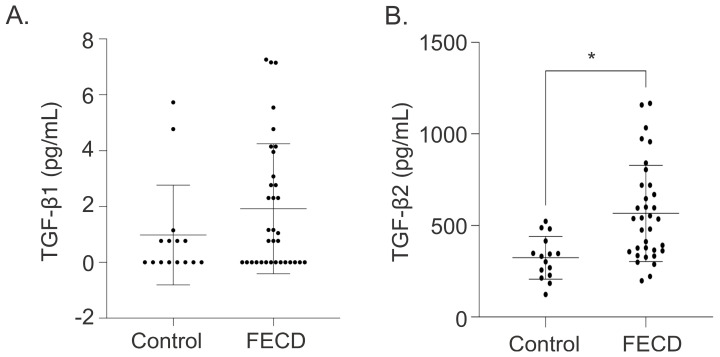
TGF-β levels in aqueous humor of FECD and control patients. Detection of (**A**) TGF-β1 and (**B**) TGF-β2 levels (mean ± SD) in aqueous humor collected from FECD patients (*n* = 34) and cataract control patients (*n* = 15). * *p*-value < 0.05 by Mann–Whitney *U* test. SD = standard deviation.

Aqueous humor (AH) was collected from 34 phakic patients undergoing Descemet’s membrane endothelial keratoplasty (DMEK) or DSO combined with cataract extraction and intraocular lens (CE-IOL) insertion with a history of FECD and 15 phakic patients with no ocular history other than cataract undergoing CE-IOL insertion only (control group). The mean age of the FECD cohort was significantly younger than that of the control cohort (67.09 ± 6.40 years vs. 73.07 ± 11.50 years, *p*-value < 0.05), and the FECD cohort was composed of 76.5% females, compared to 60.0% in the control group (Table 1). TGF-β2 levels were significantly elevated in the AH of FECD patients compared to controls (566.43 ± 263.32 pg/mL vs. 323.83 ± 116.74 pg/mL, *p*-value < 0.05; Table 1, Figure 1). However, no statistically significant difference in TGF-β1 levels was found between FECD patients and controls (1.92 ± 2.33 pg/mL vs. 0.98 ± 1.79 pg/mL, *p*-value > 0.05; Table 1, Figure 1).

### 2.2. RNA Sequencing Identifies EMT Pathway Activation in FECD CECs Treated with TGF-β

**Figure 2:**
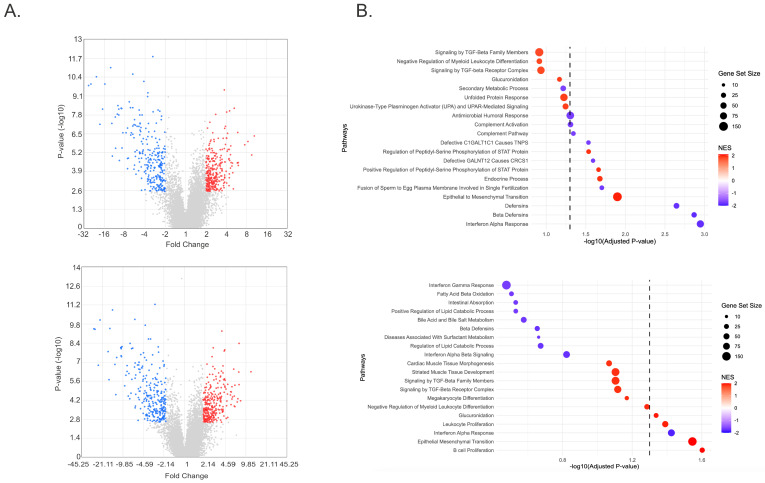
Differentially expressed genes and enriched pathways following TGF-β stimulation in FECD CECs. (**A**) Volcano plot of DEGs for TGF-β1 (**top**) and TGF-β2 (**bottom**). DEGs are based on FC <−2 and >2 with an FDR of <0.05. Red indicates DEGs that are upregulated in TGF-β group compared to control group. Blue indicates DEGs that are downregulated in TGF-β group compared to control group. (**B**) Top 10 positive and negative pathways enriched following TGF-β1 (**top**) and TGF-β2 (**bottom**) treatment in FECD CECs. The dashed line displays the adjusted *p*-value cut-off of <0.05. The size of dots indicates the number of genes represented in each pathway. The color represents the normalized enrichment score (NES), red representing a positive enrichment score and blue representing a negative enrichment score.

Although only TGF-β2 was significantly elevated in the AH of FECD patients in our cohort, others have reported elevated levels of TGF-β1 in FECD corneas [41]. Therefore, to investigate the effects of TGF-β signaling in FECD, we treated FECD CECs (FECD-SVF5-54F) with either TGF-β1 or TGF-β2 and performed bulk RNA sequencing (RNA-seq). We identified 548 differentially expressed genes (DEGs), with 289 upregulated DEGs and 259 downregulated DEGs with TGF-β1 compared to control (Figure 2A, top; Table S1). Alternatively, with TGF-β2, we identified 550 DEGs, with 292 upregulated and 258 downregulated DEGs (Figure 2A, bottom; Table S2). Of the DEGs between TGF-β1 and TGF-β2, 444 DEGs were common, 104 DEGs were unique to TGF-β1, and 106 DEGs were unique to TGF-β2 (Tables S1 and S2). Using Gene Set Enrichment Analysis (GSEA), 13 gene sets were significantly enriched for TGF-β1 using a false discovery rate (FDR) of <5% (Figure 2B, top). Of the 13 gene sets, 4 gene sets had a positive normalized enrichment score (NES), and 9 gene sets had a negative NES. Furthermore, with TGF-β2, only five gene sets were significantly enriched, four gene sets with a positive NES and only one gene set with a negative NES, using an FDR cut-off of <5% (Figure 2B, bottom). Interestingly, EMT was the most positively enriched pathway (largest positive NES) for both TGF-β1 and TGF-β2 and encompassed the most genes (149 genes) from our gene list (Table S3). With the identification of EMT as a highly enriched pathway in FECD CECs, we furthered our investigation by looking into the role of TGF-β in endothelial-to-mesenchymal transition (EndoMT) in FECD.

### 2.3. TGF-β Increases EMT Markers and Alters Morphological Phenotype

**Figure 3:**
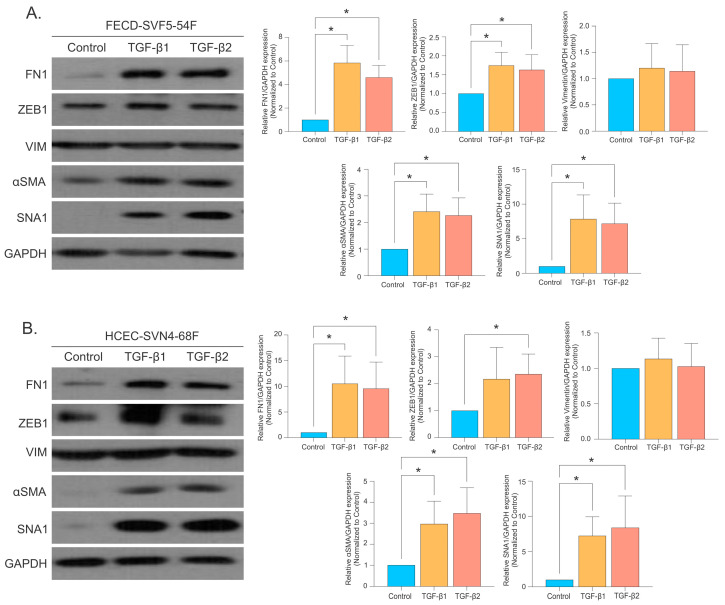
TGF-β increases EMT expression in FECD and normal control CECs. Representative images of EMT proteins and densitometry quantification (mean ± SD) for (**A**) FECD and (**B**) normal CECs after treating with TGF-β1 (10 ng/mL) or TGF-β2 (10 ng/mL). * *p*-value < 0.05 by one-way ANOVA. SD = standard deviation.

Using Western blot analysis, we probed for several EMT markers in CECs following treatment with TGF-β. We found that both TGF-β1 and TGF-β2 increased EMT markers (FN1, ZEB1, αSMA, and SNA1) in FECD CECs (Figure 3A) and in normal healthy CECs (HCECs; HCEC-SVN4-68F) (Figure 3B).

**Figure 4:**
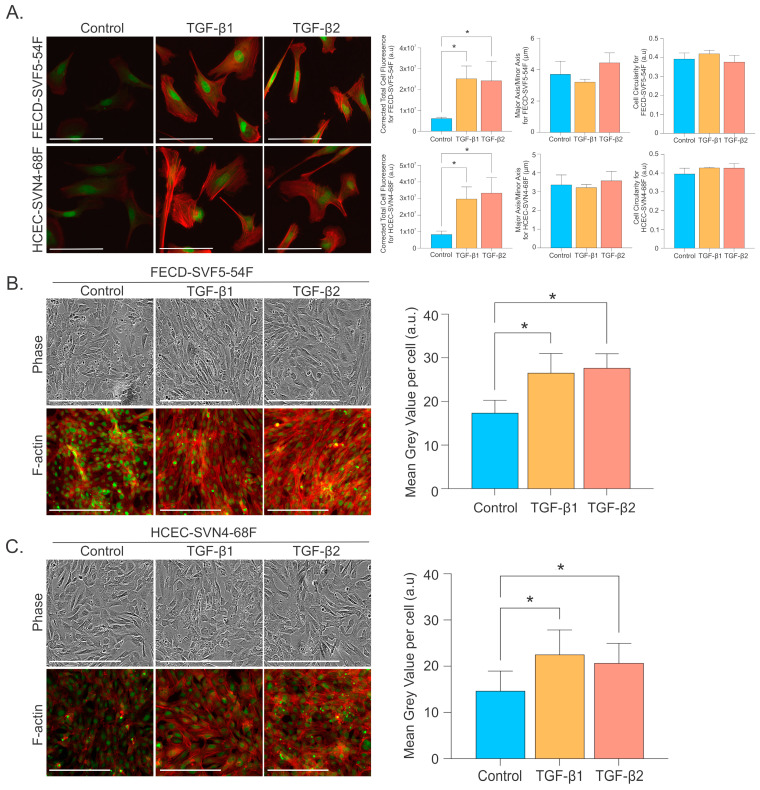
Increased filamentous-actin expression and morphological changes in CECs with TGF-β. (**A**, **left**) Representative images of filamentous-actin (F-actin) staining for FECD and normal HCECs at low confluency for control and with TGF-β1 or TGF-β2. Scale bar = 100 μm. (**A**, **right**). Quantification of corrected total cell fluorescence intensity, measurements of the major axis over minor axis of cells, and cell circularity for morphology are shown (mean ± SD) for FECD and normal HCECs with and without TGF-β1 or TGF-β2. Representative phase contrast images (scale bar = 400 μm) and filamentous actin (F-actin) staining (scale bar = 100 μm) including quantification of F-actin staining (mean ± SD) for (**B**) FECD cells and (**C**) normal HCECs. (Red = F-actin cytoskeleton staining; green = SYTOX nuclei staining.) * *p*-value < 0.05 by one-way ANOVA. SD = standard deviation.

To determine whether TGF-β alters FECD CEC morphology, we examined FECD CECs plated at low-confluency and high-confluency conditions and probed for F-action to visualize the cytoskeleton actin filaments and measured the cell shape by the ratio of the major to minor axis of cells and cell circularity. At low confluency, TGF-β induced higher F-actin staining, along with more prominent and well-organized actin filaments (Figure 4A) in FECD and normal HCECs. However, we did not detect a statistically significant difference in cell shape for the major and minor axis or cell circularity after TGF-β treatment in FECD or normal HCECs. Since the CE is composed of a monolayer of cells, we subsequently examined the effects of TGF-β at higher-confluency conditions. Consistent with low-confluency observations, TGF-β increased F-actin staining and produced dynamic, thick, parallel actin stress fiber bundles in FECD CECs (Figure 4B) and normal HCECs (Figure 4C). Additionally, we noticed a more EMT-like phenotype in FECDs with TGF-β at a higher-confluency condition (Figure 4B) that was not detected at low confluency (Figure 4A). In FECD CECs with TGF-β, cells exhibited a more elongated and fibroblastic-like phenotype (Figure 4B). Interestingly, this phenotype was not as prominent in normal HCECs (Figure 4C).

### 2.4. TGF-β Promotes Corneal Endothelial Cell Migration

**Figure 5:**
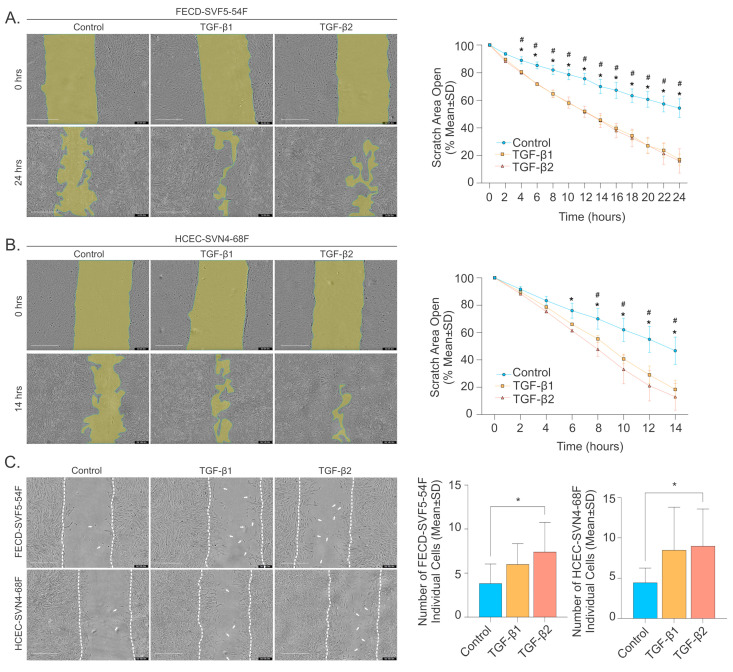
TGF-β promotes CEC migration. Representative phase contrast images of FECD (**A**, **left**) and normal HCECs (**B**, **left**) for control and TGF-β1 and TGF-β2 treatment at 0 h and 24 h or 14 h, respectively (scale bar = 400 μm). Quantification of wound closure measured by the percentage of open scratch area for FECD (**A**, **right**) and normal HCECs (**B**, **right**) over time (mean ± SD). * *p*-value < 0.05 for TGF-β2 compared to control. # *p*-value < 0.05 for TGF-β1 compared to control by two-way ANOVA. SD = standard deviation. (**C**) Representative phase contrast images (**left**) and quantification (**right**; mean ± SD) of scratch assays for FECD and normal HCECs at 10 h (scale bar = 400 μm) shows increased individual cell migration with TGF-β2. White arrows show individual cells that migrated away from the wound edge. * *p*-value < 0.05 by one-way ANOVA. SD = standard deviation.

We have previously reported that FECD CECs from ex vivo specimens displayed increased cellular migration speeds [22,23]. This, in combination with the significantly elevated levels of TGF-β2 in the AH of FECD patients, prompted us to investigate whether TGF-β could promote cellular migration in CECs. We first evaluated the wound closure rate using scratch assays and found that both TGF-β1 and TGF-β2 significantly promoted wound closure in FECD CECs compared to control (Figure 5A). In addition, we found that both TGF-β1 and TGF-β2 significantly promoted wound closure in normal CECs compared to control (Figure 5B).

To determine whether TGF-β treatment altered migration behavior by promoting individual cell movement rather than a collective monolayer spreading, we quantified the number of individual cells that migrated into the wound at 10 h post initial scratch (Figure 5C). We observed a significantly higher number of individual cells in the wound area for TGF-β2 compared to controls (7.417 ± 3.343 cells vs. 3.83 ± 2.21 cells, *p*-value < 0.05). While TGF-β1 showed a higher number of individual cells in the wound area (6.00 ± 2.34 cells vs. 3.83 ± 2.21 cells, *p*-value = 0.1325), this difference was not statistically significant (Figure 5C). In normal HCECs, a similar trend was observed, where more individual cells migrated to the wound center with TGF-β, although only the increase in TGF-β2 was statistically significant (Figure 5C, right). These findings suggest that TGF-β promotes CEC migration speed and individual cell migration into the wound area.

**Figure 6:**
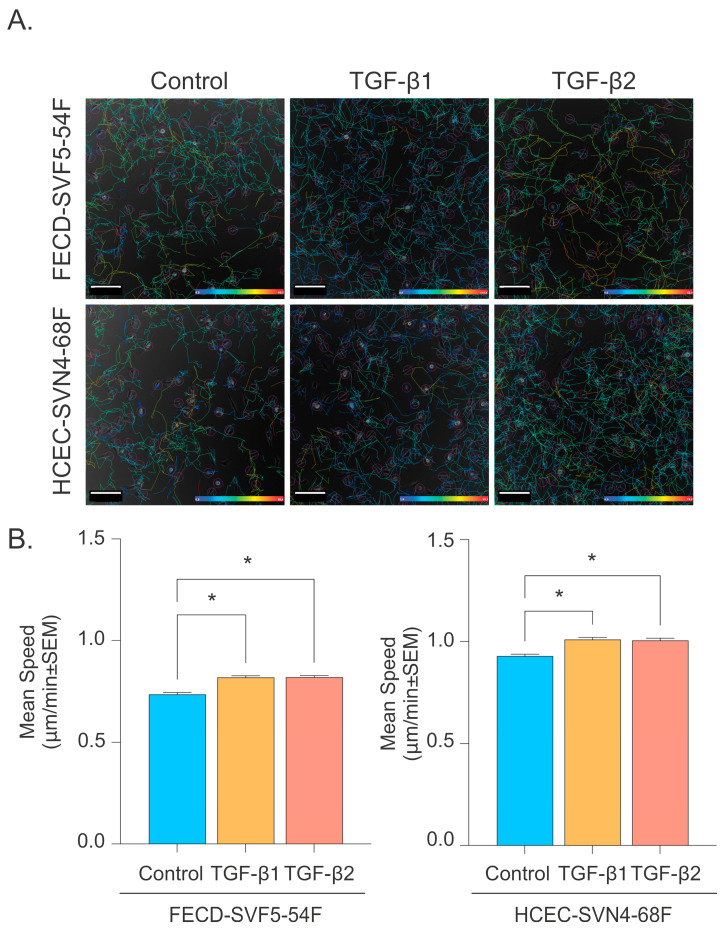
Increased individual CEC speed with TGF-β. (**A**) Representative phase contrast images for non-confluent FECD and normal HCECs. Mean migration speed visualized through color maps (scale bar = 200 μm). (**B**) Mean migration speeds of FECD and normal HCECs over 24 h for control, and with TGF-β1 or TGF-β2 treatment. * *p*-value < 0.05 by one -way ANOVA). SEM = standard error of the mean.

To determine whether TGF-β treatment would promote CEC migration when cells were not in a monolayer, we plated FECD and normal HCECs at low confluency, treated them with TGF-β1 or TGF-β2, and tracked individual cells over a 24 h period at 30 min intervals (Figure 6A). In FECD CECs, both cells treated with TGF-β1 (0.818 ± 0.456 μm/min vs. 0.737 ± 0.445 μm/min, *p*-value < 0.05) and TGF-β2 (0.819 ± 0.479 μm/min vs. 0.737 ± 0.445 μm/min, *p*-value < 0.05) had increased mean speed compared to controls (Figure 6B). Similarly, in normal HCECs, both cells treated with TGF-β1 (1.009 ± 0.482 μm/min vs. 0.928 ± 0.460 μm/min, *p*-value < 0.05) and TGF-β2 (1.005 ± 0.481 μm/min vs. 0.928 ± 0.460 μm/min, *p*-value < 0.05) had increased mean speed compared to controls (Figure 6B). There was no significant difference in mean speed between TGF-β1 and TGF-β2 treatments in FECD CEC (0.818 ± 0.456 μm/min vs. 0.819 ± 0.479 μm/min, *p*-value > 0.9999) and normal HCECs (1.009 ±0.482 μm/min vs. 1.005 ±0.483 μm/min, *p*-value = 0.9997; Figure 6B).

## 3. Discussion

We have previously reported that FECD CECs display increased migration speeds, altered migratory behavior, and cytoskeleton dysregulation in ex vivo patient specimens and cell lines [22,23]. While intrinsic cellular factors such as increased TCF4 expression can promote CEC migration [22,23], whether extrinsic factors found in the AH alters CEC migration in FECD has not been well investigated. In this study, we collected AH samples from FECD patients and control cataract patients undergoing intraocular surgery and found significantly elevated levels of TGF-β2, but not TGF-β1, in the AH of FECD patients. To investigate TGF-β-mediated effects in FECD, we performed RNA sequencing on FECD CECs treated with TGF-β1 or TGF-β2 and found that EMT was the most positively enriched pathway. We confirmed that both TGF-β1 and TGF-β2 increased EMT protein markers in FECD and normal control CECs. We also showed that both TGF-β1 and TGF-β2 altered the expression of cytoskeleton components such as F-actin in CECs, consistent with EndoMT. We also demonstrated that both TGF-β1 and TGF-β2 increased cellular migration speeds, under both low- and high-confluency conditions, and promoted more individual cell migration rather than collective cell migration in FECD CECs and normal controls. These findings suggests that elevated levels of TGF-β in the AH of FECD patients promote EndoMT and alter cellular migration behavior in CECs.

The TGF-β superfamily is a large group of diverse extracellular growth factors that signal through transmembrane serine/threonine kinase receptors and that regulate many biological processes [27,42]. All three TGF-β isoforms (TGF-β1, TGF-β2, and TGF-β3) have been found in pseudophakic and phakic FECD eyes [41,43,44]. TGF-β2 has been reported to be the predominate TGF-β isoform identified in the AH [30] and is also the predominant isoform detected following corneal wound injury [28]. Previous studies have reported differences in TGF-β isoform levels in the AH of FECD patients, where Matthaei et al. found no difference in TGF-β1 and TGF-β2 levels between phakic FECD and cataract controls but found that pseudophakic FECD eyes had elevated TGF-β1 and TGF-β2 levels, suggesting that cataract surgery alters the AH environment [41,45]. Similarly, Chychko et al. found no difference in TGF-β levels between phakic FECD and cataract control eyes [43]. In contrast, De Roo et al. found that TGF-β2 was increased in phakic FECD eyes compared to cataract controls and also in pseudophakic FECD eyes compared to pseudophakic non-FECD edematous corneas, suggesting that elevated TGF-β2 is characteristic of FECD [44]. Our results support the conclusion that TGF-β2, but not TGF-β1, is increased in the AH of phakic FECD eyes compared to cataract controls and likely contributes to FECD pathogenesis. We also found that TGF-β2 levels were higher compared to TGF-β1 levels in both FECD and control eyes. It is likely that both TGF-β1 and TGF-β2 contribute to FECD, as Okumura et al. have also found increased expression of TGF-β1 and TGF-β2 isoforms and their receptors in the corneal endothelium of FECD patients undergoing Descemet membrane endothelial keratoplasty (DMEK) compared to non-FECD donor controls [33]. However, one limitation of their study was that the lens status (phakic vs. pseudophakic) of these FECD patients was not reported.

TGF-β regulates a large range of cellular processes, including tissue fibrosis, wound healing, EndoMT, and EMT [27,28,31]. In FECD, TGF-β activation leads to the excessive accumulation of ECM proteins through EMT activating genes such as ZEB1 and SNAI1 [34]. Our RNA-seq analysis revealed that EMT was the most positively enriched pathway for both TGF-β1 and TGF-β2 pathway activation in FECD CECs. Similarly, a recent RNA-seq analysis of CECs from FECD patients revealed significant alterations in TGF-β genes, where TGF-β2 stimulation increased the expression of ECM components associated with guttae formation and EndoMT [46]. Furthermore, Nakagawa et al. demonstrated TGF-β1 expression levels vary among FECD patients based on their TCF4 trinucleotide repeat expansion status, suggesting a potential regulatory interplay between TCF4 and TGF-β signaling. Although, the TCF4 genetic status of our FECD cohort is currently unknown, our RNA seq analysis reveal that TCF4 expression is upregulated following TGF-β1 or TGF-β2 stimulation (Tables S1 and S2), further supporting an association between TCF4 and TGF-β. Additional investigations are needed to determine how various genetic mutations associated with FECD, such as TCF4 repeat expansion can influence TGF-β signaling.

TGF-β activation has also been shown to induce cell death via the UPR in FECD CECs, which can be attenuated with TGF-β inhibition [33]. Intriguingly, FECD CEC displayed an increased responsiveness to TGF-β stimulation compared to normal CECs, which was likely attributed to increased TGF-β receptor expression levels [33]. Additional studies have also demonstrated how TGF-β can influence CEC phenotype depending on whether the cells are in a proliferative phase or in a confluent maturing phase [47]. In the proliferative phase, TGF-β activation resulted in a fibroblastic CECs, whereas in the maturation phase, TGF-β activation resulted in an endothelial phenotype with functional cell junctions [47]. In our study, FECD CECs stimulated with TGF-β resulted in increased EndoMT, as detected by EndoMT protein markers and F-actin staining, under both low- and high-confluency conditions, suggesting that the TGF-β-mediated effects on FECD CECs are not dependent on cellular confluency.

More recently, CHIR99021, a GSK3 inhibitor, was shown to reverse TGF-β-mediated EndoMT and simultaneously enhance wound healing in healthy CECs, indicating a potential crosstalk between TGF-β and the Wnt/β-catenin signaling pathways [48,49]. While Wnt/β-catenin signaling pathways were not significantly enriched in our RNA seq data set, several pathway genes were differentially expressed following TGF-β stimulation in FECD CECs. Activation of Wnt/β-catenin signaling requires Wnt ligands to bind to FZD and LRP5/6 receptors [50]. *WNT5A* and *WNT9A* were significantly upregulated with TGF-β, whereas *LRP5* was downregulated with TGF-β (Tables S1 and S2) in our FECD CECs. In addition, β-catenin was modestly upregulated with TGFβ1 and TGFβ2 (1.46 and 1.49-fold, respectively) and no difference was observed for *GSK3B*. Although *WNT5A* and *WNT9A* are upregulated, their presence alone may not be sufficient to activate the Wnt/B-catenin pathway as *LRP5*, a Wnt receptor, is downregulated with TGF-β. Maurizi et al. found TGF-β1 treatment alone did not significantly activate Wnt/β-catenin signaling, but TGF-β1 and CHIR99021 together can activate canonical Wnt/β-catenin signaling [49]. Whether the transcriptional changes observed in our RNA seq data reflect functional activation of Wnt/β-catenin signaling remains to be determined. Nonetheless, our findings suggest TGF-β can modulate components in the Wnt/β-catenin pathway, implicating its potential involvement in TGF-β-mediated EndoMT in FECD CECs and warranting further investigation.

Cell migration and enlargement, rather than proliferation, is an important wound healing mechanism in the corneal endothelium [4,10,12,13,14,15,16,51]. We have previously observed that FECD CECs display increased cellular migration speeds compared to normal controls in FECD cell lines and ex vivo patient specimens [22,23]. As TGF-β is well known for inducing EMT and promoting migration, we theorized that the increase in migration speed and cytoskeleton dysregulation seen in FECD ex vivo specimens may be partly attributed to aberrant TGF-β levels in the AH. TGF-β is a key regulator of wound healing by facilitating cellular migration and proliferation to repair damaged tissues [31]. However, dysregulated TGF-β signaling can lead to fibrosis, excessive ECM deposition, and abnormal cell migration [52]. These effects are particularly relevant in the pathogenesis of FECD, where EndoMT can drive CEC migration [33,34,53,54,55,56,57]. In this study, we identified the EMT pathway as a significantly enriched pathway in FECD CECs when stimulated with TGF-β1 and TGF-β2. We observed thicker, more parallel and prominent actin stress fibers in FECD and normal CECs stimulated with TGF-β, consistent with the dynamic actin remodeling reported with TGF-β induced EMT [58,59,60]. We also detected increased cellular migration speed and more individual cell migration rather than collective migration in response to TGF-β, similar to what we have previously observed in FECD ex vivo specimens [22,23]. Taken together, we have demonstrated that there are increased TGF-β2 levels in the AH of FECD patients, which leads to EndoMT, an altered cytoskeleton, and increased migration speeds in FECD CECs. How this pro-migratory EndoMT phenotype affects in vivo cellular migration, particularly after DSO, remains to be elucidated. While TGF-β can accelerate wound healing and increase cellular migration speeds, it also promotes fibrosis, ECM deposition, and abnormal cell migration behavior [28,34,61,62,63]. Cellular migration is essential for recovery after DSO, as more normal peripheral CECs migrate centrally after stripping away the central diseased Descemet’s membrane [38,39,64,65]. As such, future studies aimed at understanding how TGF-β influences functional endothelial barrier reformation after DSO will provide valuable insight into FECD pathogenesis and may lead to novel approaches to expedite recovery after DSO.

## 4. Materials and Methods

### 4.1. Cell Culture

An immortalized FECD cell line was generated from corneal endothelial cells isolated from a 54-year-old female (FECD-SVF5-54F) undergoing endothelial keratoplasty [22]. An immortalized normal HCEC-SVN4-68F cell line was graciously provided by Dr. Ula Jurkunas at The Schepens Eye Research Institute in Boston, Massachusetts. Cells were cultured in Chen’s media consisting of Opti-MEM media (ThermoFisher Scientific, Waltham, MA, USA) supplemented with 200 mg/L CaCl_2_ (Millipore Sigma, Oakville, ON, Canada), 0.08% chondroitin sulfate (Millipore Sigma, Oakville, ON, Canada), 50 µg/mL gentamicin (ThermoFisher Scientific, Waltham, MA, USA), 1× antibiotic/antimycotic (Wisent, St. Bruno, QC, Canada), 66 µg/mL bovine pituitary extract (Gemini, West Sacramento, CA, USA), 5 ng/mL EGF (Millipore Sigma, Oakville, ON, Canada), and 8% fetal bovine serum (ThermoFisher Scientific, Waltham, MA, USA). Cell culture flasks or plates were coated with undiluted FNC coating mix (AthenaES, Baltimore, MD, USA) prior to cell seeding and incubated at 37 °C and 5% CO_2_. For functional assays, including single-cell migration, scratch assays, and F-actin morphology assay, CECs were plated in Chen’s medium overnight to allow for attachment and subsequently treated with TGF-β1 or 2 (10 ng/mL, R&D systems, Minneapolis, MN, USA) in serum-free DMEM media (Wisent, St. Bruno, QC, Canada).

### 4.2. Aqueous Humor Collection

The study was carried out according to the tenets of Declaration of Helsinki and was approved by The University of Toronto Research Ethics Board in Toronto, Canada. Inclusion criteria for study enrollment included patients with a clinical diagnosis of FECD and cataracts with no intraocular lens replacement (phakic) who were scheduled to undergo endothelial keratoplasty. Control participants were patients with a diagnosis of cataracts undergoing phacoemulsification and intraocular lens replacement. Patients with previous intraocular surgery, pre-existing ocular conditions other than FECD and cataract, or a history of chronic systemic diseases, such as fibrotic conditions, were excluded from the study. Informed consent was obtained from patients prior to sample collection at Kensington Eye Institute. Approximately 100 μL of aqueous humor was collected with a 30-gauge needle at the beginning of surgery and stored at −80 °C until use.

### 4.3. TGF-β Cytokine Analysis

Levels of TGF-β in aqueous humor samples were measured using a MILLIPLEX^®^ TGFβ 1, 2, 3 Magnetic Bead Kit (Millipore Sigma, Oakville, ON, Canada) according to the manufacturer’s protocol. Briefly, AH was diluted 1:3 and added to plates with beads overnight at 4 °C. TGF-β detection antibodies were subsequently added, followed by streptavidin–phycoerythrin. Levels of TGF-β were measured using a Luminex MAGPIX Multiplexing System (Diasorin, Austin, TX, USA). All samples were run in duplicate.

### 4.4. RNA Transcriptomics

The immortalized FECD cell line was plated in 12-well plates (Sarstedt, Montreal, QC, Canada) and incubated overnight at 37 °C. TGF-β1 or 2 (10 ng/mL, R&D systems, Minneapolis, MN, USA) in Chen’s media was added to wells for 48 h. Total RNA was extracted with PureLink RNA Mini Kit (ThermoFisher Scientific, Waltham, MA, USA) according to the manufacturer’s instructions and sent to the Genomics Core Facility at the Sunnybrook Research Institute for bulk RNA sequencing. Samples were analyzed on a Bioanalyzer RNA Pico Assay and sequencing performed with the Ion Ampliseq Transcriptome Human Assay Sequencing. Briefly, a cDNA library was constructed, and a qPCR library was quantified followed by sequencing template preparation on the Ion Chef Instrument. Sequencing was carried out with the Ion S5XL Next-Generation Sequencing and data was analyzed using Transcriptome Analysis Console (TAC) Software, version 4.0.3.14 to generate differentially expressed genes (DEGs). Genes with fold change >2 and <−2 and FDR (adjusted *p*-value) <0.05 were selected as DEGs. Gene Set Enrichment Analysis (GSEA) was performed on a ranked gene lists for TGF-β1 and TGF-β2 to identify pathways with genes enriched at the top or bottom of the ranked gene list [66]. A normalized enrichment score was generated and reflects the enrichment of the pathway in the list. Pathways with an FDR of <0.05 were considered significantly enriched.

### 4.5. Western Blot

The HCEC and FECD cell lines were seeded in 6-well plates (Sarstedt, Montreal, QC, Canada) pre-coated with undiluted FNC coating mix overnight. TGF-β1 or 2 (10 ng/mL, R&D systems, Minneapolis, MN, USA) in Chen’s media was added to wells for 48 h. Whole-cell lysates were collected in a RIPA Buffer (50 mM Tris, pH 8, 150 mM NaCl, 5 mM EDTA, pH 8, 1% NP-40, 0.5% Sodium Deoxycholate, 0.1% SDS) containing 1× protease inhibitor cocktail (Millipore Sigma, Oakville, ON, Canada) and 1 mM PMSF. Cell lysates were separated on an SDS-PAGE gel and transferred onto Amersham hybond ECL nitrocellulose membrane (Millipore Sigma, Oakville, ON, Canada). Membranes were blocked with 5% skim milk and subsequently incubated with the indicated antibodies at 4 °C overnight. Appropriate HRP-conjugated secondary antibodies were incubated for one hour at room temperature. Signals were detected using Pierce ECL Western blotting Substrate (ThermoFisher Scientific, Waltham, MA, USA) or Pierce SuperSignal West Pico Plus Chemiluminescent substrate (ThermoFisher Scientific, Waltham, MA, USA). The primary and secondary antibodies and the concentrations used were as follows: mouse anti-Fibronectin (1:1000, Santa Cruz, Dallas, TX, USA), rabbit anti-ZEB1 (1:1000, Cell Signaling, Danvers, MA, USA), rabbit anti-Snail (1:1000, Cell Signaling, Danvers, MA, USA), mouse anti-Vimentin (1:1000, Santa Cruz, Dallas, TX, USA), mouse anti-αSMA (1:1000, R&D systems, Minneapolis, MN, USA), rabbit anti-GAPDH (1:5000, Cell Signaling, Danvers, MA, USA), anti-mouse (1:10,000, ThermoFisher Scientific, Waltham, MA, USA), anti-rabbit (1:10,000, ThermoFisher Scientific, Waltham, MA, USA).

### 4.6. Individual Single-Cell Migration

The HCEC or FECD cell lines were seeded at a low density in 24-well plates (Sarstedt, Montreal, QC, Canada) pre-coated with undiluted FNC coating mix overnight. The next day, cells were pre-treated with TGF-β1 or 2 (10 ng/mL, R&D systems, Minneapolis, MN, USA) in serum-free DMEM (Wisent, St. Bruno, QC, Canada) media for 24 h before live imaging every 30 min for 24 h using a Leica DMi8 fluorescence inverted microscope (Leica Microsystems, Buffalo Grove, IL, USA) at 10× magnification. The system included a motorized 3-plate stage (Leica) with an Okolab stage top incubator (H301) for temperature (37 °C) and humidity control, connected to premixed 5% CO_2_/95% air tank. The 24-well tissue culture plate was positioned on the Okolab universal plate holder. Four separate regions of interest were imaged per well. Live imaging data was analyzed using the Trackmate plugin in Fiji, version 2.14.0, to automatically determine cell migration metrics, including mean speeds. Image sequences were imported and viewed in “default” color mode with autoscaling and a hyperstack arrangement ordered from X to T. Cell detection was performed using the Downsample LoG detector with an estimated blob diameter of 60 μm and a quality threshold of 1.746. While most cells were detected automatically, manual corrections were performed to ensure accurate cell identification. A simple linear assignment problem (LAP) tracker was used with the following parameters: frame-to-frame linking of 120 µm, max gap closing of 54 µm, and max frame gap of 2 µm. Tracks shorter than 2 frames were also excluded. Additional adjustments were made in TrackScheme to refine cell tracking. The processed tracking data were then exported for statistical analysis.

### 4.7. Scratch Assay

The HCEC or FECD cell lines were seeded in 12-well plates (Sarstedt, Montreal, QC, Canada) pre-coated with undiluted FNC coating mix overnight. TGF-β1 or 2 (10 ng/mL, R&D systems, Minneapolis, MN, USA) in serum-free DMEM media (Wisent, St. Bruno, QC, Canada) was added to wells for 24 h prior to generating a linear scratch. A P-200 pipette tip was used to generate a linear scratch. Media were removed and replaced with fresh DMEM media with TGF-β1 or 2 (10 ng/mL). Images were captured every 2 h at 10× magnification for 16–24 h with an Incucyte S3 Live-Cell Analysis system (Sartorius, Bohemia, NY, USA). Scratch assay images were analyzed using Fiji, version 2.14.0. At 10 h, the number of individual cells was counted in the wound area. Individual cells were considered cells that were fully separated from the wound edge and were not in contact with other cells along the edge of the scratch.

### 4.8. F-actin Morphology Assay

F-actin morphology assays were carried out in 24-well plates (Sarstedt, Montreal, QC, Canada) for low-confluence conditions or 12-well plates (Sarstedt, Montreal, QC, Canada) for high-confluence conditions. Cells were seeded and incubated overnight at 37 °C. Serum-free DMEM media with TGF-β1 or 2 (10 ng/mL) were added to cells for 48 h prior to F-actin staining. Wells were washed with 1× PBS, fixed with 4% paraformaldehyde for 20 min, and permeabilized with 0.3% Triton-X (Biorad, Mississauga, ON, Canada). F-actin was stained using Rhodamine Phalloidin (1:400, ThermoFisher Scientific, Waltham, MA, USA) for 1 h and counterstained with SYTOX Green Nucleic Acid Stain (ThermoFisher Scientific, Waltham, MA, USA). Images were taken with a Leica DMi8 inverted fluorescence microscope. For the low-confluence conditions, the major and minor axis of cells and the cell circularity were measured using Fiji, version 2.14.0. A circularity value of 1.0 indicates a perfect circle and a value closer to 0.0 indicates a more elongated shape. The corrected total cell fluorescence was determined by using the following calculation: (CTCF = integrated density—(Area of cell x mean fluorescence of background)). For high-confluence conditions, fluorescence intensity staining was quantified with Fiji, version 2.14.0, using the mean gray value over the total number of cells.

### 4.9. Statistical Analysis

Statistical analysis was performed using a two-tailed Mann–Whitney *U* test for the cytokine analysis. One-way or two-way analysis of variance (ANOVA) followed by Tukey’s post hoc test was performed for all other statistical analyses, unless otherwise specified. A *p*-value < 0.05 was considered statistically significant.

## Figures and Tables

**Table 1:** Demographics of aqueous humor samples.

Characteristics	Control	FECD	*p*-Value
Total, *n* (%)	15	34	
DMEK only	-	4 (11.8)	
DMEK+CE+IOL	-	25 (73.5)	
DSO+CE+IOL	-	5 (14.7)	
Age, years (Mean ± SD) [Range]	73.07 ± 11.50 [55–89]	67.09 ± 6.40 [52–77]	0.0238
Sex, n (%)			
Male	6 (40.0)	8 (23.5)	
Female	9 (60.0)	26 (76.5)	
TGF-β1, pg/mL (Mean ± SD) [Range]	0.98 ± 1.79 [0–5.73]	1.92 ± 2.33 [0–7.26]	0.1865
TGF-β2, pg/mL (Mean ± SD) [Range]	323.83 ± 116.74 [123.65–523.71]	566.43 ± 263.32 [197.96–1167.04]	0.0005

DMEK—Descemet membrane endothelial keratoplasty; DSO—Descemet Stripping Only; CE—cataract extraction; IOL—intraocular lens; SD—standard deviation.

## Data Availability

The raw data supporting the conclusions of this article will be made available by the authors on request.

## Supplementary Materials

The following supporting information can be downloaded at https://www.mdpi.com/article/10.3390/ijms26146685/s1.
