# Supplementary material for: TGF-β Promotes Endothelial-to-Mesenchymal Transition and Alters Corneal Endothelial Cell Migration in Fuchs Endothelial Corneal Dystrophy
Source: Int J Mol Sci. 2025 Jul 11;26(14):6685. doi: 10.3390/ijms26146685 (PMC12294433; doi:10.3390/ijms26146685)
Supplement: Supplementary file 1 [file ijms-26-06685-s001.zip › Table S1 DEG for TGFB1.pdf]

Table S1. List of 548 DEGs for FECD-SVF5-54F with TGF-β1

Gene Symbol	RefSeq	FC	Gene Name
**NPR3**	**NM_022463**	**27.7**	**Natriuretic Peptide Receptor C Guanylate Cyclase C Atrionatriuretic Peptide Receptor C**
**NKAIN4**	**NM_201266**	**25.45**	**Na K Transporting Atpase Interacting 4**
**PMEPA1**	**NM_002800**	**21.05**	**Prostate Transmembrane Protein Androgen Induced 1**
AMTN	NM_181847	18.73	Amelotin
ST6GAL2	NM_006307	17.05	ST6 Beta-Galactosamide Alpha-2 6-Sialyltranferase 2
NEDD9	NM_006403	15.77	Neural Precursor Cell Expressed Developmentally Down-Regulated 9
LMCD1	NM_006033	12.93	LIM And Cysteine-Rich Domains 1
LIPG	NR_026779	12.41	Lipase Endothelial
**C5orf46**	**NM_001080824**	**12.22**	**Chromosome 5 Open Reading Frame 46**
SEMA7A	NM_005063	11.02	Semaphorin 7A Gpi Membrane Anchor John Milton Hagen Blood Group
**KCNG1**	**NM_001002914**	**10.95**	**Potassium Voltage-Gated Channel Subfamily G Member 1**
SLC46A3	NM_001135919	10.33	Solute Carrier Family 46 Member 3
LAMC2	NM_005562	10.11	Laminin Gamma 2
**MAF**	**NM_002380**	**9.86**	**V-Maf Avian Musculoaponeurotic Fibrosarcoma Oncogene Homolog**
JAG1	NM_000214	9.78	Jagged 1
BHLHE40	NM_001711	9.66	Basic Helix-Loop-Helix Family Member E40
**NOX4**	**NM_001042483**	**9.08**	**NADPH Oxidase 4**
TSPAN2	NM_005725	8.85	Tetraspanin 2
**IL11**	**NM_139017**	**8.82**	**Interleukin 11**
**FSTL3**	**NM_013409**	**8.51**	**Follistatin-Like 3 Secreted Glycoprotein**
FOXS1	NM_001102371	7.68	Forkhead Box S1
DGKI	NM_004717	7.32	Diacylglycerol Kinase Iota
**LDLRAD4**	**NM_001193485**	**7.29**	**Low Density Lipoprotein Receptor Class A Domain Containing 4**
ANGPTL4	NM_212557	7.28	Angiopoietin-Like 4
PODXL	NM_025179	7.08	Podocalyxin-Like
**PTHLH**	**NM_206827**	**6.88**	**Parathyroid Hormone-Like Hormone**
TGFBI	NM_000660	6.61	Transforming Growth Factor Beta-Induced 68Kda
CSF1R	NM_000757	6.54	Colony Stimulating Factor 1 Receptor
**TFPI2**	**NM_002160**	**6.4**	**Tissue Factor Pathway Inhibitor 2**
CTGF	NM_001323	6.12	Connective Tissue Growth Factor
SIK1	NM_000602	6.12	Salt-Inducible Kinase 1
MYLK2	NM_033118	6.1	Myosin Light Chain Kinase 2
KRT17	NM_000422	6.07	Keratin 17
TAGLN	NM_152592	5.85	Transgelin
BMP6	NM_001718	5.84	Bone Morphogenetic Protein 6
**PDGFB**	**NM_007366**	**5.83**	**Platelet-Derived Growth Factor Beta Polypeptide**

HHIP	NM_022475	5.77	Hedgehog Interacting Protein
**RHOJ**	**NM_006080**	**5.75**	**Ras Homolog Family Member J**
EDN1	NM_017823	5.62	Endothelin 1
VCAN	NM_001078	5.6	Versican
CDKN2B	NM_004932	5.57	Cyclin-Dependent Kinase Inhibitor 2B P15 Inhibits CDK4
ITGA11	NM_001004439	5.45	Integrin Alpha 11
**RASL11B**	**NM_199161**	**5.31**	**RAS-Like Family 11 Member B**
SNAI1	NM_005904	5.28	Snail Family Zinc Finger 1
GPR68	NM_001177676	5.02	G Protein-Coupled Receptor 68
FOXC2	NM_005438	4.95	Forkhead Box C2 Mfh-1 Mesenchyme Forkhead 1
**CHST11**	**NM_001005735**	**4.93**	**Carbohydrate Chondroitin 4 Sulfotransferase 11**
GADD45B	NM_000812	4.88	Growth Arrest And DNA-Damage-Inducible Beta
EGR2	NM_004428	4.87	Early Growth Response 2
LRRC15	NM_002332	4.87	Leucine Rich Repeat Containing 15
MDFI	NM_005586	4.82	MyoD Family Inhibitor
**PDGFA**	**NM_001005242**	**4.82**	**Platelet-Derived Growth Factor Alpha Polypeptide**
**FLJ16779**	**NM_014845**	**4.79**	**FIG4 phosphoinositide 5-phosphatase**
INHBA	NM_002192	4.77	Inhibin Beta A
NUAK1	NM_021005	4.77	NUAK Family SNF1-Like Kinase 1
THBS1	NM_004613	4.76	Thrombospondin 1
**SPOCK1**	**NM_001013632**	**4.71**	**Sparc Osteonectin Cwcv And Kazal-Like Domains Proteoglycan Testican 1**
**JUNB**	**NM_080671**	**4.64**	**Jun B Proto-Oncogene**
S1PR5	NM_005168	4.6	Sphingosine-1-Phosphate Receptor 5
MFAP4	NM_152637	4.56	Microfibrillar-Associated Protein 4
XYLT1	NM_173570	4.54	Xylosyltransferase I
C1orf106	NM_018265	4.52	Chromosome 1 Open Reading Frame 106
**LIF**	**NR_026860**	**4.52**	**Leukemia Inhibitory Factor**
AEBP1	NM_000676	4.51	AE Binding Protein 1
ADAM19	NM_003474	4.43	ADAM Metallopeptidase Domain 19
HTR1D	NM_000864	4.4	5-Hydroxytryptamine Serotonin Receptor 1D G Protein-Coupled
LTBP2	NM_130830	4.36	Latent Transforming Growth Factor Beta Binding Protein 2
**FAP**	**NM_017938**	**4.35**	**Fibroblast Activation Protein Alpha**
MEDAG	NM_032849	4.34	Mesenteric Estrogen-Dependent Adipogenesis
PCDH1	NM_002585	4.28	Protocadherin 1
SIM2	NM_003012	4.24	Single-Minded Family Bhlh Transcription Factor 2
**SMAD7**	**NM_014465**	**4.2**	**SMAD Family Member 7**
CGB7	NM_033043	4.09	Chorionic Gonadotropin Beta Polypeptide 7
PCDH10	NM_032420	4.03	Protocadherin 10
NTM	NM_002522	4.02	Neurotrimin

**PDGFRB**	**NM_178836**	**3.99**	**Platelet-Derived Growth Factor Receptor Beta Polypeptide**
COL27A1	NM_000089	3.94	Collagen Type XXVII Alpha 1
AQP1	NM_001136540	3.91	Aquaporin 1 Colton Blood Group
**RNF150**	**NM_030666**	**3.9**	**Ring Finger Protein 150**
GDF6	NM_000557	3.87	Growth Differentiation Factor 6
FRMD6	NM_004118	3.82	FERM Domain Containing 6
HS3ST3A1	NM_001098540	3.78	Heparan Sulfate Glucosamine 3-O-Sulfotransferase 3A1
MSC	NM_002436	3.72	Musculin
NREP	NM_000435	3.7	Neuronal Regeneration Related Protein
APCDD1L-AS1	NM_153360	3.69	APCDD1L Antisense Rna 1 Head To Head
TPM1	NM_001098797	3.69	Tropomyosin 1 Alpha
AMIGO2	NM_001030010	3.67	Adhesion Molecule With Ig-Like Domain 2
LOC643650	NR_033957	3.66	long intergenic non-protein coding RNA 842 (LINC00842), long non-coding RNA
COL4A1	NM_032888	3.63	Collagen Type IV Alpha 1
CYP24A1	NM_000104	3.63	Cytochrome P450 Family 24 Subfamily A Polypeptide 1
FN1	NM_013231	3.62	Fibronectin 1
CTHRC1	NM_001901	3.6	Collagen Triple Helix Repeat Containing 1
DSP	NM_198545	3.6	Desmoplakin
HES1	NM_005524	3.59	Hes Family Bhlh Transcription Factor 1
LAPTM5	NM_006762	3.59	Lysosomal Protein Transmembrane 5
COL5A1	NM_033641	3.58	Collagen Type V Alpha 1
**SH2D2A**	**NM_001166348**	**3.58**	**SH2 Domain Containing 2A**
MMP2	NM_020300	3.57	Matrix Metallopeptidase 2 Gelatinase A 72Kda Gelatinase 72Kda Type Iv Collagenase
**BGN**	**NM_001003940**	**3.56**	**Biglycan**
**CILP**	**NM_001276**	**3.53**	**Cartilage Intermediate Layer Protein Nucleotide Pyrophosphohydrolase**
FBLN5	NM_004460	3.53	Fibulin 5
P4HA3	NM_017855	3.53	Prolyl 4-Hydroxylase Alpha Polypeptide Iii
MYO10	NM_012334	3.52	Myosin X
TMEM87B	NM_024789	3.41	Transmembrane Protein 87B
F2RL1	NM_172105	3.39	Coagulation Factor Ii Thrombin Receptor-Like 1
**MFAP2**	**NM_001531**	**3.38**	**Microfibrillar-Associated Protein 2**
STK38L	NM_020799	3.38	Serine Threonine Kinase 38 Like
ANKRD1	NM_001142446	3.37	Ankyrin Repeat Domain 1 Cardiac Muscle
KIF26B	NM_018012	3.36	Kinesin Family Member 26B
WNT5A	NM_003392	3.36	Wingless-Type Mmtv Integration Site Family Member 5A
GRB14	NM_003979	3.34	Growth Factor Receptor-Bound Protein 14
INSIG1	NM_005542	3.32	Insulin Induced Gene 1
**ODAM**	**NM_001198834**	**3.32**	**Odontogenic Ameloblast Asssociated**

TNFRSF19	NM_018647	3.32	Tumor Necrosis Factor Receptor Superfamily Member 19
PIK3IP1	NM_006474	3.31	Phosphoinositide-3-Kinase Interacting Protein 1
**SERPINE1**	**NM_004171**	**3.3**	**Serpin Peptidase Inhibitor Clade E Nexin Plasminogen Activator Inhibitor Type 1 Member 1**
GAL	NM_015675	3.28	Galanin Gmap Prepropeptide
RUNX3	NM_004292	3.28	Runt-Related Transcription Factor 3
SOX4	NM_005985	3.25	SRY Sex Determining Region Y -Box 4
**KCTD16**	**NM_002253**	**3.23**	**Potassium Channel Tetramerization Domain Containing 16**
SULT1B1	NM_015894	3.2	Sulfotransferase Family Cytosolic 1B Member 1
**PGM2L1**	**NM_020904**	**3.17**	**Phosphoglucomutase 2-Like 1**
**FLRT2**	**NR_039985**	**3.16**	**Fibronectin Leucine Rich Transmembrane Protein 2**
**GREM1**	**NM_004285**	**3.15**	**Gremlin 1 Dan Family Bmp Antagonist**
NOTCH3	NM_152864	3.11	Notch 3
COL7A1	NM_000093	3.09	Collagen Type VII Alpha 1
PLEK2	NM_001005337	3.07	Pleckstrin 2
TNFAIP6	NM_007115	3.07	Tumor Necrosis Factor Alpha-Induced Protein 6
DAAM2	NM_000782	3.06	Dishevelled Associated Activator Of Morphogenesis 2
**SLC19A2**	**NM_016354**	**3.05**	**Solute Carrier Family 19 Thiamine Transporter Member 2**
DHRS2	NM_144683	3.01	Dehydrogenase Reductase SDR Family Member 2
GJA1	NM_005110	2.99	Gap Junction Protein Alpha 1 43Kda
HEYL	NM_014571	2.97	Hes-Related Family Bhlh Transcription Factor With Yrpw Motif-Like
DACT1	NM_016651	2.96	Dishevelled-Binding Antagonist Of Beta-Catenin 1
SRPX	NM_004598	2.96	Sushi-Repeat Containing Protein X-Linked
**DRAXIN**	**NM_014787**	**2.95**	**Dorsal Inhibitory Axon Guidance Protein**
KIAA1147	NM_001080392	2.93	KIAA1147
COL18A1	NM_000494	2.92	Collagen Type XVIII Alpha 1
CA3	NM_020925	2.9	Carbonic Anhydrase Iii Muscle Specific
FHL3	NM_000800	2.89	Four And A Half Lim Domains 3
HECW2	NM_020760	2.89	HECT C2 And Ww Domain Containing E3 Ubiquitin Protein Ligase 2
ADAMTS4	NM_033274	2.88	ADAM Metallopeptidase With Thrombospondin Type 1 Motif 4
**PPAPDC1A**	**NM_022449**	**2.88**	**Phosphatidic Acid Phosphatase Type 2 Domain Containing 1A**
AKAP12	NM_016108	2.85	A Kinase PRKA Anchor Protein 12
COL1A1	NM_030582	2.84	Collagen Type I Alpha 1
UNC5B	NM_003358	2.84	Unc-5 Homolog B C. Elegans
COL4A2	NM_001845	2.78	Collagen Type IV Alpha 2
NGF	NM_002506	2.78	Nerve Growth Factor Beta Polypeptide
SCD	NM_004350	2.77	Stearoyl-CoA Desaturase Delta-9-Desaturase
WNT9A	NM_003395	2.75	Wingless-Type Mmtv Integration Site Family Member 9A

ADAM12	NM_000022	2.74	ADAM Metallopeptidase Domain 12
GALNT10	NM_015973	2.74	UDP-N-Acetyl-Alpha-D-Galactosamine Polypeptide N-Acetylgalactosaminyltransferase 10 Galnac-T10
SLAMF8	NM_003975	2.74	SLAM Family Member 8
ITGB6	NM_001144996	2.73	Integrin Beta 6
CSDC2	NM_001878	2.7	Cold Shock Domain Containing C2 Rna Binding
KAL1	NM_002229	2.69	Kallmann Syndrome 1 Sequence
MIR22HG	NM_001198695	2.68	Mir22 Host Gene Non-Protein Coding
PPP1R13L	NM_001030059	2.68	Protein Phosphatase 1 Regulatory Subunit 13 Like
SPHK1	NM_003118	2.68	Sphingosine Kinase 1
RGS16	NM_023940	2.67	Regulator Of G-Protein Signaling 16
XDH	NM_022166	2.67	Xanthine Dehydrogenase
SLC7A5	NM_003486	2.64	Solute Carrier Family 7 Amino Acid Transporter Light Chain L System Member 5
APCDD1L	NM_001159	2.61	Adenomatosis Polyposis Coli Down-Regulated 1-Like
LRIG3	NM_014646	2.6	Leucine-Rich Repeats And Immunoglobulin-Like Domains 3
**CGB5**	**NM_007186**	**2.59**	**Chorionic Gonadotropin Beta Polypeptide 5**
GPAM	NM_020918	2.59	Glycerol-3-Phosphate Acyltransferase Mitochondrial
TGM2	NM_003243	2.59	Transglutaminase 2
SKIL	NM_000199	2.58	SKI-Like Oncogene
FGF1	NM_001184866	2.57	Fibroblast Growth Factor 1 Acidic
LINC00152	NM_014988	2.57	Long Intergenic Non-Protein Coding Rna 152
PTPRK	NM_004878	2.57	Protein Tyrosine Phosphatase Receptor Type K
DUSP23	NM_001390	2.53	Dual Specificity Phosphatase 23
KDM6B	NM_020768	2.53	Lysine K -Specific Demethylase 6B
TGFB1	NM_006528	2.53	Transforming Growth Factor Beta 1
TMEM92	NM_032824	2.52	Transmembrane Protein 92
**ADA**	**NM_001613**	**2.51**	**Adenosine Deaminase**
**ATP13A2**	**NM_021732**	**2.5**	**ATPase Type 13A2**
CACHD1	NM_018398	2.5	Cache Domain Containing 1
TNS1	NM_022648	2.5	Tensin 1
KCTD11	NM_002250	2.49	Potassium Channel Tetramerization Domain Containing 11
ACTA2	NM_016234	2.48	Actin Alpha 2 Smooth Muscle Aorta
CHAC1	NM_033142	2.48	Chac Cation Transport Regulator Homolog 1 E. Coli
GPR87	NM_023915	2.48	G Protein-Coupled Receptor 87
EFNA1	NM_001955	2.47	Ephrin-A1
STK17B	NM_032528	2.46	Serine Threonine Kinase 17B
KDR	NM_001080424	2.45	Kinase Insert Domain Receptor A Type Iii Receptor Tyrosine Kinase
MATN3	NM_002381	2.45	Matrilin 3
COL10A1	NM_003632	2.43	Collagen Type X Alpha 1

ADAMTS5	NM_005099	2.42	ADAM Metallopeptidase With Thrombospondin Type 1 Motif 5
LRP12	NM_153377	2.42	Low Density Lipoprotein Receptor-Related Protein 12
BMP1	NM_006129	2.41	Bone Morphogenetic Protein 1
KLF7	NM_003709	2.41	Kruppel-Like Factor 7 Ubiquitous
TCF4	NM_003206	2.41	Transcription Factor 4
UCK2	NM_012474	2.41	Uridine-Cytidine Kinase 2
FURIN	NM_000147	2.4	Furin Paired Basic Amino Acid Cleaving Enzyme
CGB	NM_000737	2.39	Chorionic Gonadotropin Beta Polypeptide
**LRP1**	**NM_017527**	**2.39**	**Low Density Lipoprotein Receptor-Related Protein 1**
CCL20	NM_006848	2.37	Chemokine C-C Motif Ligand 20
DIXDC1	NM_004675	2.37	Dix Domain Containing 1
IL32	NM_001012718	2.37	Interleukin 32
**MLXIP**	**NM_053025**	**2.37**	**Mlx Interacting Protein**
FCRLA	NM_006329	2.35	Fc Receptor-Like A
FOSB	NM_212482	2.35	Fbj Murine Osteosarcoma Viral Oncogene Homolog B
PLXNA2	NM_014935	2.35	Plexin A2
HMOX1	NM_002133	2.34	Heme Oxygenase Decycling 1
LOC728392	NM_001162371	2.34	Uncharacterized LOC728392
IGFBP7	NM_001553	2.33	Insulin-Like Growth Factor Binding Protein 7
POSTN	NM_020182	2.33	Periostin Osteoblast Specific Factor
BMF	NM_182962	2.31	Bcl2 Modifying Factor
IVNS1ABP	NM_006469	2.31	Influenza Virus NS1A Binding Protein
JARID2	NM_004973	2.31	Jumonji At Rich Interactive Domain 2
**NRP2**	**NM_001142599**	**2.31**	**Neuropilin 2**
ARFGAP1	NM_000044	2.3	ADP-Ribosylation Factor Gtpase Activating Protein 1
CST6	NM_005211	2.3	Cystatin E M
NCOR2	NM_006312	2.3	Nuclear Receptor Corepressor 2
PALLD	NM_005446	2.3	Palladin Cytoskeletal Associated Protein
PKP1	NM_001172438	2.29	Plakophilin 1 Ectodermal Dysplasia Skin Fragility Syndrome
HMCN1	NM_031935	2.28	Hemicentin 1
PLA2R1	NM_007350	2.28	Phospholipase A2 Receptor 1 180KDa
RALB	NM_004163	2.28	V-Ral Simian Leukemia Viral Oncogene Homolog B
SIGLEC15	NM_004155	2.28	Sialic Acid Binding Ig-Like Lectin 15
SLC29A1	NM_015482	2.28	Solute Carrier Family 29 Equilibrative Nucleoside Transporter Member 1
**CDH11**	**NM_003914**	**2.26**	**Cadherin 11 Type 2 OB-Cadherin Osteoblast**
TNC	NM_001193451	2.26	Tenascin C
**TSC22D3**	**NM_000379**	**2.26**	**TSC22 Domain Family Member 3**
VEGFA	NM_004385	2.24	Vascular Endothelial Growth Factor A
INHBE	NM_031479	2.23	Inhibin Beta E

PDPN	NM_002607	2.22	Podoplanin
RASGRP1	NM_002889	2.22	RAS Guanyl Releasing Protein 1 Calcium And Dag-Regulated
DLC1	NM_033425	2.21	Deleted In Liver Cancer 1
GFPT2	NM_001001557	2.21	Glutamine-Fructose-6-Phosphate Transaminase 2
GPR183	NM_004951	2.21	G Protein-Coupled Receptor 183
**ITGAV**	**NM_004791**	**2.21**	**Integrin Alpha V**
ROBO4	NM_002928	2.21	Roundabout Axon Guidance Receptor Homolog 4 Drosophila
CXXC5	NM_002994	2.2	CXXC Finger Protein 5
**NLRP1**	**NM_001134231**	**2.2**	**NLR Family Pyrin Domain Containing 1**
NT5DC2	NM_001204375	2.19	5P-Nucleotidase Domain Containing 2
**NUPR1**	**NM_031458**	**2.18**	**Nuclear Protein Transcriptional Regulator 1**
TSPAN15	NM_012339	2.17	Tetraspanin 15
**CACNA2D3**	**NM_001217**	**2.16**	**Calcium Channel Voltage-Dependent Alpha 2 Delta Subunit 3**
PRR5L	NM_006663	2.13	Proline Rich 5 Like
PHLDB1	NM_002609	2.11	Pleckstrin Homology-Like Domain Family B Member 1
MARCH4	NM_018050	2.1	Membrane-Associated Ring Finger C3HC4 4 E3 Ubiquitin Protein Ligase
SLC22A3	NM_001145098	2.1	Solute Carrier Family 22 Organic Cation Transporter Member 3
COL1A2	NM_000088	2.09	Collagen Type I Alpha 2
KCNE4	NM_000216	2.09	Potassium Voltage-Gated Channel Isk-Related Family Member 4
MEOX1	NM_004527	2.08	Mesenchyme Homeobox 1
ARHGAP31	NM_004815	2.07	Rho GTPase Activating Protein 31
DDB2	NM_001352	2.07	Damage-Specific Dna Binding Protein 2 48KDa
BCL11A	NM_022893	2.06	B-Cell CLL Lymphoma 11A Zinc Finger Protein
TCTEX1D4	NM_001083962	2.06	Tctex1 Domain Containing 4
TAGLN2	NM_003186	2.05	Transgelin 2
LIMS1	NM_002309	2.04	Lim And Senescent Cell Antigen-Like Domains 1
ZNF469	NM_001127464	2.04	Zinc Finger Protein 469
SPARC	NM_003107	2.03	Secreted Protein Acidic Cysteine-Rich Osteonectin
CNN1	NM_020872	2.02	Calponin 1 Basic Smooth Muscle
**GRAP**	**NM_004490**	**2.02**	**GRB2-Related Adaptor Protein**
TUFT1	NM_020127	2.02	Tuftelin 1
P4HA2	NM_002538	2	Prolyl 4-Hydroxylase Alpha Polypeptide Ii
ENO2	NM_004433	-2	Enolase 2 Gamma Neuronal
COL8A1	NM_000094	-2.01	Collagen Type VIII Alpha 1
EPHX4	NM_006209	-2.01	Epoxide Hydrolase 4
RND3	NM_145654	-2.01	Rho Family Gtpase 3
ARHGAP20	NM_001657	-2.02	Rho GTPase Activating Protein 20
**LPIN2**	**NM_203422**	**-2.02**	**Lipin 2**

SLC1A2	NM_173354	-2.02	Solute Carrier Family 1 Glial High Affinity Glutamate Transporter Member 2
**AVPI1**	**NM_005581**	**-2.03**	**Arginine Vasopressin-Induced 1**
BTC	NM_001729	-2.03	Betacellulin
MAL2	NM_203403	-2.03	Mal T-Cell Differentiation Protein 2 Gene Pseudogene
MATN2	NM_020814	-2.03	Matrilin 2
**SIGIRR**	**NM_080669**	**-2.03**	**Single Immunoglobulin And Toll-Interleukin 1 Receptor Tir Domain**
SLC15A3	NM_014521	-2.03	Solute Carrier Family 15 Oligopeptide Transporter Member 3
STAMBPL1	NM_012446	-2.03	STAM Binding Protein-Like 1
PLD6	NM_001144758	-2.04	Phospholipase D Family Member 6
CCNA1	NM_004591	-2.05	Cyclin A1
GALNT5	NM_024642	-2.05	UDP-N-Acetyl-Alpha-D-Galactosamine Polypeptide N-Acetylgalactosaminyltransferase 5 Galnac-T5
SSBP2	NM_016950	-2.05	Single-Stranded Dna Binding Protein 2
CXCL5	NM_002090	-2.06	Chemokine C-X-C Motif Ligand 5
DNAJC6	NM_182643	-2.06	Dnaj Hsp40 Homolog Subfamily C Member 6
MUC1	NM_145015	-2.06	Mucin 1 Cell Surface Associated
**SGK1**	**NM_021977**	**-2.06**	**Serum Glucocorticoid Regulated Kinase 1**
ZFYVE28	NM_024697	-2.06	Zinc Finger FYVE Domain Containing 28
AIG1	NM_032717	-2.07	Androgen-Induced 1
APOL1	NR_034147	-2.07	Apolipoprotein L 1
AR	NM_001651	-2.07	Androgen Receptor
FIG4	NM_004468	-2.08	Fig4 Homolog Sac1 Lipid Phosphatase Domain Containing S. Cerevisiae
**ACSL5**	**NM_020039**	**-2.1**	**Acyl-CoA Synthetase Long-Chain Family Member 5**
**CUEDC1**	**NM_000396**	**-2.1**	**Cue Domain Containing 1**
ELF3	NM_000399	-2.1	E74-Like Factor 3 Ets Domain Transcription Factor Epithelial-Specific
FAM115C	NM_012306	-2.1	Family With Sequence Similarity 115 Member C
PAMR1	NM_182904	-2.1	Peptidase Domain Containing Associated With Muscle Regeneration 1
PSMB9	NM_001166161	-2.1	Proteasome Prosome Macropain Subunit Beta Type 9
MALL	NM_014400	-2.11	Mal T-Cell Differentiation Protein-Like
SERPINB1	NM_003004	-2.11	Serpin Peptidase Inhibitor Clade B Ovalbumin Member 1
SMAD3	NM_005902	-2.11	SMAD Family Member 3
**ACBD7**	**NM_001039844**	**-2.12**	**Acyl-CoA Binding Domain Containing 7**
**ADCK2**	**NM_007038**	**-2.12**	**AarF Domain Containing Kinase 2**
CTSO	NM_001334	-2.12	Cathepsin O
RASL11A	NM_004585	-2.12	RAS-Like Family 11 Member A
AKR1C2	NM_001353	-2.13	Aldo-Keto Reductase Family 1 Member C2
GLRX	NM_000165	-2.13	Glutaredoxin Thioltransferase
IGFBP6	NM_002178	-2.13	Insulin-Like Growth Factor Binding Protein 6

LIMCH1	NM_181481	-2.13	Lim And Calponin Homology Domains 1
SLC46A1	NM_001078176	-2.13	Solute Carrier Family 46 Folate Transporter Member 1
SLC4A4	NM_001134742	-2.14	Solute Carrier Family 4 Sodium Bicarbonate Cotransporter Member 4
ALDH3B1	NM_205845	-2.15	Aldehyde Dehydrogenase 3 Family Member B1
ITGBL1	NM_002210	-2.15	Integrin Beta-Like 1 With EGF-Like Repeat Domains
**RARRES1**	**NM_020724**	**-2.15**	**Retinoic Acid Receptor Responder Tazarotene Induced 1**
**SLCO4A1**	**NM_015000**	**-2.15**	**Solute Carrier Organic Anion Transporter Family Member 4A1**
MYLK	NM_016132	-2.16	Myosin Light Chain Kinase
ASIC1	NM_032199	-2.17	Acid-Sensing Proton-Gated Ion Channel 1
BST2	NM_004335	-2.17	Bone Marrow Stromal Cell Antigen 2
ENPP2	NM_001975	-2.17	Ectonucleotide Pyrophosphatase Phosphodiesterase 2
TMTC1	NM_001168215	-2.17	Transmembrane And Tetratricopeptide Repeat Containing 1
MOXD1	NR_028502	-2.18	Monooxygenase DBH-Like 1
**PLEKHA2**	**NM_015869**	**-2.18**	**Pleckstrin Homology Domain Containing Family A Phosphoinositide Binding Specific Member 2**
TMEM180	NM_015444	-2.18	Transmembrane Protein 180
RAB17	NM_001135648	-2.19	RAB17 Member RAS Oncogene Family
RDM1	NM_005739	-2.19	RAD52 Motif 1
CTSK	NM_138455	-2.2	Cathepsin K
DPP4	NM_001935	-2.2	Dipeptidyl-Peptidase 4
KITLG	NM_000899	-2.2	KIT Ligand
MYEF2	NM_001178046	-2.2	Myelin Expression Factor 2
PLEKHA4	NM_000930	-2.2	Pleckstrin Homology Domain Containing Family A Phosphoinositide Binding Specific Member 4
PPP1R9A	NM_002705	-2.2	Protein Phosphatase 1 Regulatory Subunit 9A
**UGCG**	**NM_001118890**	**-2.2**	**UDP-Glucose Ceramide Glucosyltransferase**
**ANGPT1**	**NM_001146**	**-2.21**	**Angiopoietin 1**
AOX1	NM_007193	-2.21	Aldehyde Oxidase 1
AXL	NM_001684	-2.21	AXL Receptor Tyrosine Kinase
CCDC106	NM_033292	-2.21	Coiled-Coil Domain Containing 106
G0S2	NM_002569	-2.21	G0 G1Switch 2
PARP9	NM_001166108	-2.21	Poly ADP-Ribose Polymerase Family Member 9
**ALDH3A1**	**NM_001135168**	**-2.22**	**Aldehyde Dehydrogenase 3 Family Member A1**
COL12A1	NM_004370	-2.22	Collagen Type XII Alpha 1
**ITGA7**	**NM_000888**	**-2.22**	**Integrin Alpha 7**
MR1	NM_014938	-2.22	Major Histocompatibility Complex Class I-Related
CXCL3	NM_002089	-2.24	Chemokine C-X-C Motif Ligand 3
MITF	NM_032623	-2.24	Microphthalmia-Associated Transcription Factor
PKP2	NM_173582	-2.24	Plakophilin 2
SARM1	NM_021136	-2.24	Sterile Alpha And Tir Motif Containing 1

DHRS3	NM_004753	-2.25	Dehydrogenase Reductase SDR Family Member 3
LOC643723	NR_038845	-2.25	LYPLAL1 divergent transcript
MX1	NM_005098	-2.25	Myxovirus Influenza Virus Resistance 1 Interferon-Inducible Protein P78 Mouse
CEP250	NM_004936	-2.26	Centrosomal Protein 250kDa
**DAPK1**	**NM_001201427**	**-2.26**	**Death-Associated Protein Kinase 1**
**ZNF385D**	**NM_001127380**	**-2.26**	**Zinc Finger Protein 385D**
CA11	NM_032310	-2.27	Carbonic Anhydrase Xi
HSBP1L1	NM_006042	-2.27	Heat Shock Factor Binding Protein 1-Like 1
**NRP1**	**NM_002562**	**-2.27**	**Neuropilin 1**
TM4SF1	NM_024817	-2.27	Transmembrane 4 L Six Family Member 1
ADORA2B	NM_001124	-2.28	Adenosine A2B Receptor
FAM129A	NM_001130025	-2.29	Family With Sequence Similarity 129 Member A
**FOXRED2**	**NM_004473**	**-2.29**	**FAD-Dependent Oxidoreductase Domain Containing 2**
SVIL	NM_021738	-2.29	Supervillin
ZDHHC23	NM_020972	-2.29	Zinc Finger DHHC-Type Containing 23
**ACADS**	**NM_000017**	**-2.3**	**Acyl-CoA Dehydrogenase C-2 To C-3 Short Chain**
EYA4	NM_006495	-2.3	Eyes Absent Homolog 4 Drosophila
SLC25A10	NM_020125	-2.3	Solute Carrier Family 25 Mitochondrial Carrier Dicarboxylate Transporter Member 10
**PDE4DIP**	**NM_052880**	**-2.31**	**Phosphodiesterase 4D Interacting Protein**
PPARG	NM_005397	-2.31	Peroxisome Proliferator-Activated Receptor Gamma
**MGST1**	**NM_001204287**	**-2.32**	**Microsomal Glutathione S-Transferase 1**
RARRES3	NM_206963	-2.32	Retinoic Acid Receptor Responder Tazarotene Induced 3
SLC7A4	NM_004173	-2.32	Solute Carrier Family 7 Member 4
**LRRN4CL**	**NM_052886**	**-2.33**	**LRRN4 C-Terminal Like**
**NMT2**	**NM_001144058**	**-2.33**	**N-Myristoyltransferase 2**
**RBM47**	**NM_015077**	**-2.33**	**RNA Binding Motif Protein 47**
TNFRSF6B	NM_003823	-2.33	Tumor Necrosis Factor Receptor Superfamily Member 6B Decoy
GAS2L3	NM_014568	-2.34	Growth Arrest-Specific 2 Like 3
FOSL1	NM_006732	-2.36	Fos-Like Antigen 1
LY6K	NM_018334	-2.36	Lymphocyte Antigen 6 Complex Locus K
RTN1	NM_020663	-2.36	Reticulon 1
NRG2	NM_016931	-2.38	Neuregulin 2
RIN1	NM_001098634	-2.38	Ras And Rab Interactor 1
**LYPD3**	**NM_001168465**	**-2.39**	**LY6 Plaur Domain Containing 3**
MYO5C	NM_018728	-2.39	Myosin Vc
MPP1	NM_198159	-2.4	Membrane Protein Palmitoylated 1 55KDa
SERPINB9	NM_152754	-2.41	Serpin Peptidase Inhibitor Clade B Ovalbumin Member 9
C3	NM_000064	-2.42	Complement Component 3
CHEK2	NM_024111	-2.42	Checkpoint Kinase 2

FOXE1	NM_005251	-2.42	Forkhead Box E1 Thyroid Transcription Factor 2
**SGSH**	**NM_012140**	**-2.42**	**N-Sulfoglucosamine Sulfohydrolase**
ANXA1	NM_173505	-2.44	Annexin A1
ARID5B	NM_020754	-2.44	AT Rich Interactive Domain 5B Mrf1-Like
B3GALNT1	NM_021913	-2.44	Beta-1 3-N-Acetylgalactosaminyltransferase 1 Globoside Blood Group
MET	NM_000245	-2.44	Met Proto-Oncogene
FUCA1	NM_005860	-2.45	Fucosidase Alpha-L- 1 Tissue
IL31RA	NM_001562	-2.45	Interleukin 31 Receptor A
PEG10	NM_002608	-2.46	Paternally Expressed 10
**SAA1**	**NM_001143676**	**-2.46**	**Serum Amyloid A1**
**LURAP1L**	**NM_001130915**	**-2.47**	**Leucine Rich Adaptor Protein 1-Like**
FLJ22447	NR_024389	-2.48	LOC100192386 (FLJ16779), long non-coding RNA
LMNA	NM_014583	-2.48	Lamin A C
PPL	NM_003713	-2.48	Periplakin
RAPGEF1	NM_002881	-2.48	Rap Guanine Nucleotide Exchange Factor Gef 1
TRIOBP	NM_198057	-2.48	TRIO And F-Actin Binding Protein
BEX1	NM_018476	-2.49	Brain Expressed X-Linked 1
RAB27B	NM_002852	-2.49	RAB27B Member RAS Oncogene Family
SLC26A11	NM_006996	-2.5	Solute Carrier Family 26 Anion Exchanger Member 11
ATP2B4	NM_032827	-2.51	ATPase Ca Transporting Plasma Membrane 4
GSE1	NM_013372	-2.51	Gse1 Coiled-Coil Protein
TMEFF2	NM_001184723	-2.51	Transmembrane Protein With EGF-Like And Two Follistatin-Like Domains 2
MEST	NM_002402	-2.52	Mesoderm Specific Transcript
**SYNE3**	**NM_016192**	**-2.52**	**Spectrin Repeat Containing Nuclear Envelope Family Member 3**
CSF1	NM_014460	-2.53	Colony Stimulating Factor 1 Macrophage
SLC22A23	NM_005069	-2.53	Solute Carrier Family 22 Member 23
KRT80	NM_182507	-2.56	Keratin 80
PPAP2B	NM_007257	-2.56	Phosphatidic Acid Phosphatase Type 2B
GMPR	NM_006877	-2.57	Guanosine Monophosphate Reductase
DTNA	NM_004415	-2.58	Dystrobrevin Alpha
RARRES2	NM_198679	-2.58	Retinoic Acid Receptor Responder Tazarotene Induced 2
**LINC00341**	**NR_033931**	**-2.6**	**Long Intergenic Non-Protein Coding Rna 341**
CHI3L1	NM_018413	-2.61	Chitinase 3-Like 1 Cartilage Glycoprotein-39
DBP	NM_004938	-2.61	D Site Of Albumin Promoter Albumin D-Box Binding Protein
COL17A1	NM_001130103	-2.62	Collagen Type XVII Alpha 1
CPT1A	NM_016352	-2.62	Carnitine Palmitoyltransferase 1A Liver
METTL7B	NM_014033	-2.62	Methyltransferase Like 7B
**DHRS13**	**NM_000107**	**-2.63**	**Dehydrogenase Reductase SDR Family Member 13**
LPAR1	NM_001401	-2.63	Lysophosphatidic Acid Receptor 1

OAS1	NM_013982	-2.63	2P-5P-Oligoadenylate Synthetase 1 40 46KDa
TSPAN18	NM_130783	-2.63	Tetraspanin 18
CCDC85B	NM_013301	-2.64	Coiled-Coil Domain Containing 85B
KLHL4	NM_019117	-2.64	Kelch-Like Family Member 4
TMCO4	NM_014220	-2.65	Transmembrane And Coiled-Coil Domains 4
UBA7	NM_003335	-2.65	Ubiquitin-Like Modifier Activating Enzyme 7
CDC42EP3	NM_006449	-2.66	CDC42 Effector Protein Rho Gtpase Binding 3
OCLN	NM_003873	-2.66	Occludin
**DIO2**	**NM_182908**	**-2.67**	**Deiodinase Iodothyronine Type Ii**
SAA2	NM_019055	-2.67	Serum Amyloid A2
BNIP3	NM_004052	-2.68	BCL2 Adenovirus E1B 19Kda Interacting Protein 3
COL4A6	NM_001846	-2.68	Collagen Type IV Alpha 6
BIRC3	NM_003670	-2.72	Baculoviral Iap Repeat Containing 3
TRABD2A	NM_001018020	-2.72	TraB Domain Containing 2A
FAM70A	NM_080829	-2.74	RIPOR family member 3
HGF	NM_000601	-2.74	Hepatocyte Growth Factor Hepapoietin A Scatter Factor
SPOCK3	NM_182965	-2.74	Sparc Osteonectin Cwcv And Kazal-Like Domains Proteoglycan Testican 3
CARD16	NM_052889	-2.76	Caspase Recruitment Domain Family Member 16
HHEX	NM_002729	-2.76	Hematopoietically Expressed Homeobox
VWA5A	NM_014622	-2.76	Von Willebrand Factor A Domain Containing 5A
CD200	NM_001004196	-2.77	CD200 Molecule
P2RX7	NM_016816	-2.78	Purinergic Receptor P2X Ligand-Gated Ion Channel 7
**SEMA3A**	**NM_213602**	**-2.78**	**Sema Domain Immunoglobulin Domain Ig Short Basic Domain Secreted Semaphorin 3A**
AKR1B10	NM_005100	-2.79	Aldo-Keto Reductase Family 1 Member B10 Aldose Reductase
**HPSE**	**NM_001136180**	**-2.79**	**Heparanase**
CFB	NM_001710	-2.8	Complement Factor B
MANSC1	NM_005360	-2.8	MANSC Domain Containing 1
AQP5	NM_001185060	-2.81	Aquaporin 5
MAMSTR	NM_177964	-2.82	MEF2 Activating Motif And Sap Domain Containing Transcriptional Regulator
PNMA2	NM_001129728	-2.82	Paraneoplastic Ma Antigen 2
KCNN4	NM_002237	-2.84	Potassium Intermediate Small Conductance Calcium-Activated Channel Subfamily N Member 4
KHK	NM_006488	-2.84	Ketohexokinase Fructokinase
SLC48A1	NM_017842	-2.84	Solute Carrier Family 48 Heme Transporter Member 1
FAIM2	NM_001993	-2.85	Fas Apoptotic Inhibitory Molecule 2
IFI35	NM_005533	-2.85	Interferon-Induced Protein 35
BCAM	NM_033169	-2.86	Basal Cell Adhesion Molecule Lutheran Blood Group
C9orf89	NM_206966	-2.87	Chromosome 9 Open Reading Frame 89
CLDN3	NM_005602	-2.87	Claudin 3

MAP7D2	NM_005434	-2.87	MAP7 Domain Containing 2
PDGFRA	NM_018945	-2.88	Platelet-Derived Growth Factor Receptor Alpha Polypeptide
NXN	NM_001142476	-2.89	Nucleoredoxin
THSD4	NM_003246	-2.89	Thrombospondin Type I Domain Containing 4
IKZF2	NM_016260	-2.92	IKAROS Family Zinc Finger 2 Helios
PLEKHA6	NM_016445	-2.92	Pleckstrin Homology Domain Containing Family A Member 6
TMEM158	NM_181719	-2.93	Transmembrane Protein 158 Gene Pseudogene
CYP1B1	NM_016463	-2.96	Cytochrome P450 Family 1 Subfamily B Polypeptide 1
LINC00473	NR_024204	-2.99	Long Intergenic Non-Protein Coding Rna 473
LAMA3	NM_198129	-3	Laminin Alpha 3
SH3BP4	NM_002575	-3.01	SH3-Domain Binding Protein 4
**CNTN3**	**NM_005127**	**-3.03**	**Contactin 3 Plasmacytoma Associated**
PDE7B	NM_032961	-3.03	Phosphodiesterase 7B
F3	NM_005242	-3.1	Coagulation Factor Iii Thromboplastin Tissue Factor
ATP8B1	NM_022089	-3.13	ATPase Aminophospholipid Transporter Class I Type 8B Member 1
ANKRD29	NM_014391	-3.14	Ankyrin Repeat Domain 29
KYNU	NM_003937	-3.18	Kynureninase
GCHFR	NM_004120	-3.2	GTP Cyclohydrolase I Feedback Regulator
CA12	NM_001218	-3.22	Carbonic Anhydrase Xii
ZNF395	NM_018660	-3.22	Zinc Finger Protein 395
**AREG**	**NM_020809**	**-3.25**	**Amphiregulin**
ARHGAP29	NM_175609	-3.25	Rho GTPase Activating Protein 29
**PLAT**	**NM_006475**	**-3.25**	**Plasminogen Activator Tissue**
STMN3	NM_004226	-3.25	Stathmin-Like 3
TSLP	NM_033035	-3.27	Thymic Stromal Lymphopoietin
AKR1C1	NM_020299	-3.28	Aldo-Keto Reductase Family 1 Member C1
P2RX6	NM_014840	-3.28	Purinergic Receptor P2X Ligand-Gated Ion Channel 6
GPR39	NM_001508	-3.3	G Protein-Coupled Receptor 39
FRZB	NM_152330	-3.31	Frizzled-Related Protein
NPTX1	NM_033004	-3.35	Neuronal Pentraxin I
**CNTNAP1**	**NM_001299**	**-3.36**	**Contactin Associated Protein 1**
**TOX2**	**NM_000638**	**-3.39**	**TOX High Mobility Group Box Family Member 2**
ANK1	NM_139314	-3.43	Ankyrin 1 Erythrocytic
BMPER	NM_133468	-3.43	Bmp Binding Endothelial Regulator
IL18	NM_000641	-3.44	Interleukin 18 Interferon-Gamma-Inducing Factor
**GBP2**	**NM_174942**	**-3.45**	**Guanylate Binding Protein 2 Interferon-Inducible**
BMP4	NM_130851	-3.46	Bone Morphogenetic Protein 4
**CASP1**	**NM_005181**	**-3.46**	**Caspase 1 Apoptosis-Related Cysteine Peptidase**
CLEC2B	NM_001306	-3.46	C-Type Lectin Domain Family 2 Member B

KIAA1199	NM_018689	-3.47	KIAA1199
VTN	NM_001171623	-3.47	Vitronectin
GALNT12	NM_198321	-3.51	UDP-N-Acetyl-Alpha-D-Galactosamine Polypeptide N-Acetylgalactosaminyltransferase 12 Galnac-T12
MRGPRF	NM_004530	-3.6	MAS-Related Gpr Member F
SLCO2B1	NM_007256	-3.62	Solute Carrier Organic Anion Transporter Family Member 2B1
LRRN3	NM_013437	-3.63	Leucine Rich Repeat Neuronal 3
NDP	NM_000266	-3.67	Norrie Disease Pseudoglioma
LYPD6B	NM_000428	-3.73	LY6 Plaur Domain Containing 6B
**ADM**	**NM_052853**	**-3.76**	**Adrenomedullin**
PHLDA1	NM_006206	-3.76	Pleckstrin Homology-Like Domain Family A Member 1
CRABP2	NM_001876	-3.77	Cellular Retinoic Acid Binding Protein 2
GABRB1	NM_015714	-3.78	Gamma-Aminobutyric Acid GABA A Receptor Beta 1
H6PD	NM_014615	-3.78	Hexose-6-Phosphate Dehydrogenase Glucose 1-Dehydrogenase
LOC152742	NM_170707	-3.78	lamin A/C (LMNA)
SEMA3D	NM_001166215	-3.78	Sema Domain Immunoglobulin Domain Ig Short Basic Domain Secreted Semaphorin 3D
IL6R	NM_000565	-3.83	Interleukin 6 Receptor
PBX1	NM_001001991	-3.86	Pre-B-Cell Leukemia Homeobox 1
CXADR	NM_017949	-3.89	Coxsackie Virus And Adenovirus Receptor
**METTL7A**	**NM_015529**	**-3.91**	**Methyltransferase Like 7A**
**ATOH8**	**NM_005603**	**-3.98**	**Atonal Homolog 8 Drosophila**
NR2F2	NM_004808	-4.01	Nuclear Receptor Subfamily 2 Group F Member 2
C10orf54	NM_022153	-4.05	Chromosome 10 Open Reading Frame 54
PLEKHG4	NM_021623	-4.05	Pleckstrin Homology Domain Containing Family G With Rhogef Domain Member 4
MGARP	NM_002403	-4.06	Mitochondria-Localized Glutamic Acid-Rich Protein
**TM4SF18**	**NM_001039141**	**-4.14**	**Transmembrane 4 L Six Family Member 18**
MYPN	NM_032578	-4.18	Myopalladin
CDH6	NM_001797	-4.27	Cadherin 6 Type 2 K-Cadherin Fetal Kidney
TCF21	NM_003564	-4.34	Transcription Factor 21
FST	NM_001463	-4.38	Follistatin
ABCG2	NM_004827	-4.41	ATP-Binding Cassette Sub-Family G White Member 2
SFRP1	NM_003612	-4.44	Secreted Frizzled-Related Protein 1
CXCL2	NM_001338	-4.45	Chemokine C-X-C Motif Ligand 2
DIRAS3	NM_013989	-4.45	Diras Family Gtp-Binding Ras-Like 3
HPCAL1	NM_002149	-4.48	Hippocalcin-Like 1
KRT19	NM_002276	-4.49	Keratin 19
PTX3	NM_198965	-4.6	Pentraxin 3 Long
**GPRC5A**	**NM_006613**	**-4.61**	**G Protein-Coupled Receptor Family C Group 5 Member A**

AGPAT9	NM_001129	-4.68	1-Acylglycerol-3-Phosphate O-Acyltransferase 9
CLDN11	NM_003613	-4.7	Claudin 11
FAM65C	NM_052966	-4.87	Family With Sequence Similarity 65 Member C
TGFBR3	NM_000358	-4.95	Transforming Growth Factor Beta Receptor Iii
VCAM1	NM_170744	-4.97	Vascular Cell Adhesion Molecule 1
**EVI2A**	**NM_173567**	**-5.1**	**Ecotropic Viral Integration Site 2A**
ANXA10	NM_000700	-5.27	Annexin A10
**EVI2B**	**NM_014210**	**-5.86**	**Ecotropic Viral Integration Site 2B**
**GDF5**	**NM_005258**	**-5.88**	**Growth Differentiation Factor 5**
**COL13A1**	**NM_000493**	**-5.9**	**Collagen Type XIII Alpha 1**
PTGES	NM_024841	-6.04	Prostaglandin E Synthase
TNFRSF1B	NM_001066	-6.05	Tumor Necrosis Factor Receptor Superfamily Member 1B
**SECTM1**	**NM_021805**	**-7.53**	**Secreted And Transmembrane 1**
**SERPINB2**	**NM_016582**	**-8.38**	**Serpin Peptidase Inhibitor Clade B Ovalbumin Member 2**
COLEC12	NM_020351	-9.53	Collectin Sub-Family Member 12
CPA4	NM_130386	-10.46	Carboxypeptidase A4

* Fold Change relative to Control

Bolded genes are unique DEGs for TGF-β1
