# Supplementary material for: TGF-β Promotes Endothelial-to-Mesenchymal Transition and Alters Corneal Endothelial Cell Migration in Fuchs Endothelial Corneal Dystrophy
Source: Int J Mol Sci. 2025 Jul 11;26(14):6685. doi: 10.3390/ijms26146685 (PMC12294433; doi:10.3390/ijms26146685)
Supplement: Supplementary file 1 [file ijms-26-06685-s001.zip › Table S2 DEG for TGFB2.pdf]

Table S2. List of 550 DEGs for FECD-SVF5-54F with TGF-β2

Gene Symbol	RefSeq	FC	Gene Name
NPR3	NM_001204375	29.04	Natriuretic Peptide Receptor C Guanylate Cyclase C Atrionatriuretic Peptide Receptor C
NKAIN4	NM_152864	27.79	Na K Transporting ATPase Interacting 4
AMTN	NM_212557	24.61	Amelotin
PMEPA1	NM_020182	23.06	Prostate Transmembrane Protein Androgen Induced 1
ST6GAL2	NM_032528	17.2	St6 Beta-Galactosamide Alpha-2 6-Sialyltranferase 2
KCNG1	NM_002237	16.6	Potassium Voltage-Gated Channel Subfamily G Member 1
NEDD9	NM_006403	15.96	Neural Precursor Cell Expressed Developmentally Down-Regulated 9
LMCD1	NM_014583	14.46	LIM And Cysteine-Rich Domains 1
LIPG	NM_006033	13.3	Lipase Endothelial
C5orf46	NM_206966	12.85	Chromosome 5 Open Reading Frame 46
SEMA7A	NM_003612	12.39	Semaphorin 7A GPI Membrane Anchor John Milton Hagen Blood Group
LAMC2	NM_005562	10.63	Laminin Gamma 2
JAG1	NM_000214	10.54	Jagged 1
MAF	NM_005360	10.53	V-Maf Avian Musculoaponeurotic Fibrosarcoma Oncogene Homolog
NOX4	NM_016931	10.52	NADPH Oxidase 4
SLC46A3	NM_001135919	10.36	Solute Carrier Family 46 Member 3
TSPAN2	NM_005725	10.14	Tetraspanin 2
IL11	NM_000641	9.24	Interleukin 11
FSTL3	NM_005860	8.82	Follistatin-Like 3 Secreted Glycoprotein
FOXS1	NM_004118	8.47	Forkhead Box S1
LDLRAD4	NM_181481	8.44	Low Density Lipoprotein Receptor Class A Domain Containing 4
ANGPTL4	NM_139314	8.04	Angiopoietin-Like 4
TFPI2	NM_006528	7.88	Tissue Factor Pathway Inhibitor 2
BHLHE40	NM_003670	7.66	Basic Helix-Loop-Helix Family Member E40
PTHLH	NM_198965	7.54	Parathyroid Hormone-Like Hormone
PODXL	NM_005397	7.28	Podocalyxin-Like
TGFBI	NM_000358	7.13	Transforming Growth Factor Beta-Induced 68KDa
PDGFB	NM_002608	7.06	Platelet-Derived Growth Factor Beta Polypeptide
CTGF	NM_001901	6.81	Connective Tissue Growth Factor
KRT17	NM_000422	6.39	Keratin 17
SIK1	NM_173354	6.32	Salt-Inducible Kinase 1
CSF1R	NM_005211	6.28	Colony Stimulating Factor 1 Receptor
SNAI1	NM_005985	6.26	Snail Family Zinc Finger 1
TAGLN	NM_003186	6.25	Transgelin
DGKI	NM_004717	6.22	Diacylglycerol Kinase Iota
EGR2	NM_000399	6.15	Early Growth Response 2

MYLK2	NM_033118	6.15	Myosin Light Chain Kinase 2
BMP6	NM_001718	5.71	Bone Morphogenetic Protein 6
**VSTM4**	**NM_001031746**	**5.6**	**V-Set And Transmembrane Domain Containing 4**
FLJ16779	NR_024389	5.53	Uncharacterized LOC100192386
BGN	NM_001711	5.52	Biglycan
NUAK1	NM_014840	5.48	NUAK Family SNF1-Like Kinase 1
CHST11	NM_018413	5.46	Carbohydrate Chondroitin 4 Sulfotransferase 11
HHIP	NM_022475	5.4	Hedgehog Interacting Protein
CGB7	NM_033142	5.36	Chorionic Gonadotropin Beta Polypeptide 7
RASL11B	NM_023940	5.35	RAS-Like Family 11 Member B
AEBP1	NM_001129	5.25	AE Binding Protein 1
CDKN2B	NM_004936	5.25	Cyclin-Dependent Kinase Inhibitor 2B P15 Inhibits CDK4
JUNB	NM_002229	5.24	Jun B Proto-Oncogene
ITGA11	NM_001004439	5.13	Integrin Alpha 11
PDGFRB	NM_002609	5.11	Platelet-Derived Growth Factor Receptor Beta Polypeptide
PDGFA	NM_002607	5.09	Platelet-Derived Growth Factor Alpha Polypeptide
THBS1	NM_003246	4.89	Thrombospondin 1
S1PR5	NM_001166215	4.84	Sphingosine-1-Phosphate Receptor 5
MFAP4	NM_001198695	4.83	Microfibrillar-Associated Protein 4
INHBA	NM_002192	4.76	Inhibin Beta A
DSP	NM_004415	4.7	Desmoplakin
MDFI	NM_005586	4.66	MyoD Family Inhibitor
NOTCH3	NM_000435	4.65	Notch 3
PCDH1	NM_032420	4.6	Protocadherin 1
GADD45B	NM_015675	4.58	Growth Arrest And DNA-Damage-Inducible Beta
FOXC2	NM_005251	4.52	Forkhead Box C2 MFH-1 Mesenchyme Forkhead 1
EDN1	NM_001955	4.47	Endothelin 1
NREP	NM_001142476	4.44	Neuronal Regeneration Related Protein
ADAM19	NM_033274	4.38	ADAM Metallopeptidase Domain 19
XYLT1	NM_022166	4.38	Xylosyltransferase I
RHOJ	NM_020663	4.37	Ras Homolog Family Member J
SPOCK1	NM_004598	4.35	Sparc Osteonectin Cwcv And Kazal-Like Domains Proteoglycan Testican 1
SMAD7	NM_005904	4.32	SMAD Family Member 7
FRMD6	NM_152330	4.28	FERM Domain Containing 6
CTHRC1	NM_138455	4.24	Collagen Triple Helix Repeat Containing 1
RUNX3	NM_004350	4.22	Runt-Related Transcription Factor 3
CILP	NM_003613	4.16	Cartilage Intermediate Layer Protein Nucleotide Pyrophosphohydrolase
HTR1D	NM_000864	4.16	5-Hydroxytryptamine Serotonin Receptor 1D G Protein-Coupled
CYP24A1	NM_000782	4.14	Cytochrome P450 Family 24 Subfamily A Polypeptide 1

MEDAG	NM_032849	4.12	Mesenteric Estrogen-Dependent Adipogenesis
VCAN	NM_004385	4.12	Versican
MMP2	NM_004530	4.11	Matrix Metallopeptidase 2 Gelatinase A 72KDa Gelatinase 72KDa Type Iv Collagenase
GDF6	NM_001001557	4.09	Growth Differentiation Factor 6
C1orf106	NM_018265	4.07	Chromosome 1 Open Reading Frame 106
FAP	NM_004460	4.07	Fibroblast Activation Protein Alpha
FN1	NM_212482	4.07	Fibronectin 1
UNC5B	NM_170744	4.05	Unc-5 Homolog B C. Elegans
KIF26B	NM_018012	4.03	Kinesin Family Member 26B
LRRC15	NM_130830	4.02	Leucine Rich Repeat Containing 15
ODAM	NM_017855	3.96	Odontogenic Ameloblast Asssociated
AMIGO2	NM_181847	3.93	Adhesion Molecule With Ig-Like Domain 2
LIF	NM_002309	3.93	Leukemia Inhibitory Factor
SIM2	NM_005069	3.89	Single-Minded Family bHLH Transcription Factor 2
TNFAIP6	NM_007115	3.88	Tumor Necrosis Factor Alpha-Induced Protein 6
COL27A1	NM_032888	3.84	Collagen Type XXVII Alpha 1
LOC643650	NR_033957	3.81	Long Intergenic non-protein coding RNA 842
TPM1	NM_001018020	3.79	Tropomyosin 1 Alpha
HES1	NM_005524	3.78	Hes Family bHLH Transcription Factor 1
MYO10	NM_012334	3.74	Myosin X
LAPTM5	NM_006762	3.71	Lysosomal Protein Transmembrane 5
SERPINE1	NM_000602	3.7	Serpin Peptidase Inhibitor Clade E Nexin Plasminogen Activator Inhibitor Type 1 Member 1
ANKRD1	NM_014391	3.69	Ankyrin Repeat Domain 1 Cardiac Muscle
ADAMTS4	NM_005099	3.68	ADAM Metallopeptidase With Thrombospondin Type 1 Motif 4
MSC	NM_005098	3.67	Musculin
SH2D2A	NM_003975	3.67	SH2 Domain Containing 2A
COL5A1	NM_000093	3.65	Collagen Type V Alpha 1
PCDH10	NM_032961	3.65	Protocadherin 10
**CRLF1**	**NM_004750**	**3.57**	**Cytokine Receptor-Like Factor 1**
GAL	NM_015973	3.56	Galanin GMAP Prepropeptide
MFAP2	NM_002403	3.55	Microfibrillar-Associated Protein 2
DACT1	NM_016651	3.54	Dishevelled-Binding Antagonist Of Beta-Catenin 1
HS3ST3A1	NM_006042	3.54	Heparan Sulfate Glucosamine 3-O-Sulfotransferase 3A1
SOX4	NM_003107	3.53	Sry Sex Determining Region Y -Box 4
COL4A1	NM_001845	3.52	Collagen Type IV Alpha 1
COL7A1	NM_000094	3.51	Collagen Type VII Alpha 1
TNFRSF19	NM_018647	3.5	Tumor Necrosis Factor Receptor Superfamily Member 19
GREM1	NM_013372	3.49	Gremlin 1 DAN Family Bmp Antagonist
WNT5A	NM_003392	3.42	Wingless-Type MMTV Integration Site Family Member 5A

FHL3	NM_004468	3.38	Four And A Half Lim Domains 3
LTBP2	NM_000428	3.36	Latent Transforming Growth Factor Beta Binding Protein 2
**OLFM2**	**NM_058164**	**3.3**	**Olfactomedin 2**
APCDD1L-AS1	NR_034147	3.29	APCDD1L Antisense RNA 1 Head To Head
COL18A1	NM_030582	3.29	Collagen Type XVIII Alpha 1
GPR87	NM_023915	3.27	G Protein-Coupled Receptor 87
INHBE	NM_031479	3.26	Inhibin Beta E
SLC19A2	NM_006996	3.26	Solute Carrier Family 19 Thiamine Transporter Member 2
AQP1	NM_001185060	3.23	Aquaporin 1 Colton Blood Group
APCDD1L	NM_153360	3.19	Adenomatosis Polyposis Coli Down-Regulated 1-Like
HEYL	NM_014571	3.19	Hes-Related Family bHLH Transcription Factor With YRPW Motif-Like
P4HA3	NM_182904	3.18	Prolyl 4-Hydroxylase Alpha Polypeptide Iii
PPAPDC1A	NM_001030059	3.18	Phosphatidic Acid Phosphatase Type 2 Domain Containing 1A
HMCN1	NM_031935	3.15	Hemicentin 1
COL1A1	NM_000088	3.13	Collagen Type I Alpha 1
WNT9A	NM_003395	3.12	Wingless-Type MMTV Integration Site Family Member 9A
FBLN5	NM_006329	3.11	Fibulin 5
DRAXIN	NM_198545	3.09	Dorsal Inhibitory Axon Guidance Protein
FLRT2	NM_013231	3.07	Fibronectin Leucine Rich Transmembrane Protein 2
SRPX	NM_006307	3.05	Sushi-Repeat Containing Protein X-Linked
AKAP12	NM_005100	3.04	A Kinase PRKA Anchor Protein 12
GPR68	NM_001177676	3.04	G Protein-Coupled Receptor 68
LINC00152	NR_024204	3.04	Long Intergenic Non-Protein Coding RNA 152
KAL1	NM_000216	3.02	Kallmann Syndrome 1 Sequence
ROBO4	NM_019055	3.02	Roundabout Axon Guidance Receptor Homolog 4 Drosophila
PLEK2	NM_016445	3	Pleckstrin 2
SLC7A5	NM_003486	2.94	Solute Carrier Family 7 Amino Acid Transporter Light Chain L System Member 5
TMEM87B	NM_032824	2.94	Transmembrane Protein 87B
RASGRP1	NM_005739	2.93	RAS Guanyl Releasing Protein 1 Calcium And Dag-Regulated
DIXDC1	NM_033425	2.9	DIX Domain Containing 1
PPP1R13L	NM_006663	2.9	Protein Phosphatase 1 Regulatory Subunit 13 Like
PGM2L1	NM_173582	2.89	Phosphoglucomutase 2-Like 1
RGS16	NM_002928	2.89	Regulator Of G-Protein Signaling 16
SPHK1	NM_182965	2.88	Sphingosine Kinase 1
SLAMF8	NM_020125	2.87	SLAM Family Member 8
**MYH16**	**NR_002147**	**2.83**	**Myosin Heavy Chain 16 Pseudogene**
CACNA2D3	NM_018398	2.83	Calcium Channel Voltage-Dependent Alpha 2 Delta Subunit 3
F2RL1	NM_005242	2.79	Coagulation Factor Ii Thrombin Receptor-Like 1
COL4A2	NM_001846	2.78	Collagen Type IV Alpha 2

DUSP23	NM_017823	2.77	Dual Specificity Phosphatase 23
NLRP1	NM_033004	2.77	NKR Family Pyrin Domain Containing 1
ADAM12	NM_003474	2.76	ADAM Metallopeptidase Domain 12
KCTD16	NM_020768	2.76	Potassium Channel Tetramerization Domain Containing 16
LOC728392	NM_001162371	2.75	uncharacterized LOC728392
NCOR2	NM_006312	2.74	Nuclear Receptor Corepressor 2
FGF1	NM_000800	2.73	Fibroblast Growth Factor 1 Acidic
GPAM	NM_020918	2.73	Glycerol-3-Phosphate Acyltransferase Mitochondrial
MATN3	NM_002381	2.73	Matrilin 3
MLXIP	NM_014938	2.73	MLX Interacting Protein
STK17B	NM_004226	2.73	Serine Threonine Kinase 17B
IL32	NM_001012718	2.72	Interleukin 32
PALLD	NM_001166108	2.68	Palladin Cytoskeletal Associated Protein
**MAP3K4**	**NM_005922**	**2.65**	**Mitogen-Activated Protein Kinase Kinase Kinase 4**
TGFB1	NM_000660	2.65	Transforming Growth Factor Beta 1
UCK2	NM_012474	2.65	Uridine-Cytidine Kinase 2
CGB5	NM_033043	2.64	Chorionic Gonadotropin Beta Polypeptide 5
PTPRK	NM_001135648	2.61	Protein Tyrosine Phosphatase Receptor Type K
LRP12	NM_013437	2.6	Low Density Lipoprotein Receptor-Related Protein 12
SKIL	NM_001145098	2.6	SKI-Like Oncogene
FOSB	NM_006732	2.57	FBJ Murine Osteosarcoma Viral Oncogene Homolog B
ATP13A2	NM_022089	2.56	Atpase Type 13A2
HECW2	NM_020760	2.56	HECT C2 And WW Domain Containing E3 Ubiquitin Protein Ligase 2
TMEM92	NM_001168215	2.56	Transmembrane Protein 92
CDH11	NM_001797	2.54	Cadherin 11 Type 2 OB-Cadherin Osteoblast
CSDC2	NM_014460	2.54	Cold Shock Domain Containing C2 RNA Binding
GJA1	NM_000165	2.53	Gap Junction Protein Alpha 1 43KDa
GALNT10	NM_198321	2.5	UDP-N-Acetyl-Alpha-D-Galactosamine Polypeptide N-Acetylgalactosaminyltransferase 10 Galnac-T10
SCD	NM_005063	2.5	Stearoyl-CoA Desaturase Delta-9-Desaturase
INSIG1	NM_005542	2.49	Insulin Induced Gene 1
LRP1	NM_002332	2.49	Low Density Lipoprotein Receptor-Related Protein 1
**LOC728643**	**NR_003277**	**2.48**	**heterogeneous nuclear ribonucleoprotein A1 pseudogene 33**
**MIR181A2HG**	**NR_038975**	**2.48**	**MIR181A2 Host Gene Non-Protein Coding**
CACHD1	NM_020925	2.48	Cache Domain Containing 1
KIAA1147	NM_001080392	2.47	KIAA1147
CCL20	NM_004591	2.46	Chemokine C-C Motif Ligand 20
**RXFP3**	**NM_016568**	**2.45**	**Relaxin Insulin-Like Family Peptide Receptor 3**
CHAC1	NM_024111	2.44	ChaC Cation Transport Regulator Homolog 1 E. Coli
EFNA1	NM_004428	2.43	Ephrin-A1

LRIG3	NM_153377	2.43	Leucine-Rich Repeats And Immunoglobulin-Like Domains 3
PRR5L	NM_024841	2.39	Proline Rich 5 Like
TSPAN15	NM_012339	2.39	Tetraspanin 15
FURIN	NM_002569	2.38	Furin Paired Basic Amino Acid Cleaving Enzyme
TCF4	NM_001083962	2.38	Transcription Factor 4
TGM2	NM_004613	2.38	Transglutaminase 2
BMP1	NM_006129	2.37	Bone Morphogenetic Protein 1
FCRLA	NM_001184866	2.37	Fc Receptor-Like A
CGB	NM_000737	2.35	Chorionic Gonadotropin Beta Polypeptide
PKP1	NM_001005337	2.35	Plakophilin 1 Ectodermal Dysplasia Skin Fragility Syndrome
TNS1	NM_022648	2.35	Tensin 1
IGFBP7	NM_001553	2.33	Insulin-Like Growth Factor Binding Protein 7
MIR22HG	NR_028502	2.33	MIR22 Host Gene Non-Protein Coding
**FOXP1**	**NM_032682**	**2.32**	**Forkhead Box P1**
**HES4**	**NM_021170**	**2.32**	**Hes Family bHLH Transcription Factor 4**
PLXNA2	NM_025179	2.32	Plexin A2
ADA	NM_000022	2.3	Adenosine Deaminase
IVNS1ABP	NM_006469	2.3	Influenza Virus Ns1A Binding Protein
COL1A2	NM_000089	2.28	Collagen Type I Alpha 2
**KRT79**	**NM_175834**	**2.27**	**Keratin 79**
MARCH4	NM_020814	2.26	Membrane-Associated Ring Finger C3HC4 4 E3 Ubiquitin Protein Ligase
NGF	NM_002506	2.26	Nerve Growth Factor Beta Polypeptide
**SYNPO**	**NM_001166208**	**2.25**	**Synaptopodin**
CXXC5	NM_016463	2.22	CXXC Finger Protein 5
DLC1	NM_182643	2.22	Deleted In Liver Cancer 1
GPR183	NM_004951	2.22	G Protein-Coupled Receptor 183
KLF7	NM_003709	2.22	Kruppel-Like Factor 7 Ubiquitous
SLC29A1	NM_001078176	2.22	Solute Carrier Family 29 Equilibrative Nucleoside Transporter Member 1
BCL11A	NM_022893	2.2	B-Cell CLL Lymphoma 11A Zinc Finger Protein
GFPT2	NM_005110	2.2	Glutamine-Fructose-6-Phosphate Transaminase 2
PDPN	NM_006474	2.2	Podoplanin
JARID2	NM_004973	2.19	Jumonji At Rich Interactive Domain 2
**CREB3L1**	**NM_052854**	**2.18**	**cAMP Responsive Element Binding Protein 3-Like 1**
**FERMT1**	**NM_017671**	**2.18**	**Fermitin Family Member 1**
**TP53I3**	**NM_004881**	**2.18**	**Tumor Protein P53 Inducible Protein 3**
**DLX1**	**NM_178120**	**2.17**	**Distal-Less Homeobox 1**
**CSRNP1**	**NM_033027**	**2.15**	**Cysteine-Serine-Rich Nuclear Protein 1**
**SDC2**	**NM_002998**	**2.15**	**Syndecan 2**
ITGAV	NM_002210	2.15	Integrin Alpha V

MEOX1	NM_004527	2.15	Mesenchyme Homeobox 1
**LHX1**	**NM_005568**	**2.14**	**LIM Homeobox 1**
**DDX26B**	**NM_182540**	**2.13**	**DEAD H Asp-Glu-Ala-Asp His Box Polypeptide 26B**
HMOX1	NM_002133	2.13	Heme Oxygenase Decycling 1
**GLI2**	**NM_005270**	**2.12**	**GLI Family Zinc Finger 2**
**GRIN2D**	**NM_000836**	**2.12**	**Glutamate Receptor Ionotropic N-Methyl D-Aspartate 2D**
ARFGAP1	NM_175609	2.12	ADP-Ribosylation Factor Gtpase Activating Protein 1
KDM6B	NM_001080424	2.1	Lysine K -Specific Demethylase 6B
RALB	NM_002881	2.1	V-Ral Simian Leukemia Viral Oncogene Homolog B
ZNF469	NM_001127464	2.09	Zinc Finger Protein 469
**RIN2**	**NM_001242581**	**2.08**	**Ras And Rab Interactor 2**
**HMGCS1**	**NM_002130**	**2.07**	**3-Hydroxy-3-Methylglutaryl-CoA Synthase 1 Soluble**
**SNAI2**	**NM_003068**	**2.07**	**Snail Family Zinc Finger 2**
ARHGAP31	NM_020754	2.07	Rho GTPase Activating Protein 31
CST6	NM_001323	2.07	Cystatin E M
TAGLN2	NM_003564	2.07	Transgelin 2
**PLXNA4**	**NM_020911**	**2.06**	**Plexin A4**
**ZSWIM4**	**NM_023072**	**2.06**	**Zinc Finger SWIM-Type Containing 4**
VEGFA	NM_001171623	2.06	Vascular Endothelial Growth Factor A
PHLDB1	NM_001144758	2.05	Pleckstrin Homology-Like Domain Family B Member 1
SPARC	NM_003118	2.03	Secreted Protein Acidic Cysteine-Rich Osteonectin
**RHOB**	**NM_004040**	**2.02**	**Ras Homolog Family Member B**
TSC22D3	NM_198057	2.02	TSC22 Domain Family Member 3
**HOMER2**	**NM_199330**	**2.01**	**Homer Homolog 2 Drosophila**
**TMEM45A**	**NM_018004**	**2.01**	**Transmembrane Protein 45A**
TUFT1	NM_020127	2.01	Tuftelin 1
**MBOAT2**	**NM_138799**	**2**	**Membrane Bound O-Acyltransferase Domain Containing 2**
**STC1**	**NM_003155**	**-2.01**	**Stanniocalcin 1**
**LYPD6**	**NM_001195685**	**-2.02**	**LY6 PLAUR Domain Containing 6**
**MAP1A**	**NM_002373**	**-2.02**	**Microtubule-Associated Protein 1A**
**CD14**	**NM_000591**	**-2.03**	**CD14 Molecule**
**HEXDC**	**NM_173620**	**-2.04**	**Hexosaminidase Glycosyl Hydrolase Family 20 Catalytic Domain Containing**
**PRKAA2**	**NM_006252**	**-2.04**	**Protein Kinase Amp-Activated Alpha 2 Catalytic Subunit**
DTNA	NM_001390	-2.04	Dystrobrevin Alpha
**ANKRD5**	**NM_198798**	**-2.05**	**Ankyrin Repeat and EF-hand Domain Containing 1**
**APH1B**	**NM_031301**	**-2.05**	**APH1B Gamma Secretase Subunit**
**KIF7**	**NM_198525**	**-2.05**	**Kinesin Family Member 7**
MYEF2	NM_016132	-2.05	Myelin Expression Factor 2
UGCG	NM_003358	-2.05	UDP-Glucose Ceramide Glucosyltransferase
CCDC106	NM_013301	-2.06	Coiled-Coil Domain Containing 106

**PHF10**	**NM_018288**	**-2.07**	**PHD Finger Protein 10**
**PTPN6**	**NM_080548**	**-2.07**	**Protein Tyrosine Phosphatase Non-Receptor Type 6**
**GAMT**	**NM_138924**	**-2.08**	**Guanidinoacetate N-Methyltransferase**
**PCBD1**	**NM_000281**	**-2.08**	**Pterin-4 Alpha-Carbinolamine Dehydratase Dimerization Cofactor Of Hepatocyte Nuclear Factor 1 Alpha**
**EID3**	**NM_001008394**	**-2.09**	**EP300 Interacting Inhibitor Of Differentiation 3**
**MTSS1L**	**NM_138383**	**-2.09**	**Metastasis Suppressor 1-Like**
NMT2	NM_004808	-2.09	N-Myristoyltransferase 2
**C1orf56**	**NM_017860**	**-2.1**	**Chromosome 1 Open Reading Frame 56**
**EMP1**	**NM_001423**	**-2.1**	**Epithelial Membrane Protein 1**
NRG2	NM_013982	-2.1	Neuregulin 2
AXL	NM_021913	-2.11	AXL Receptor Tyrosine Kinase
**MAP3K5**	**NM_005923**	**-2.13**	**Mitogen-Activated Protein Kinase Kinase Kinase 5**
FOXRED2	NM_001102371	-2.13	FAD-Dependent Oxidoreductase Domain Containing 2
**MOCOS**	**NM_017947**	**-2.14**	**Molybdenum Cofactor Sulfurase**
**PAM**	**NM_000919**	**-2.14**	**Peptidylglycine Alpha-Amidating Monooxygenase**
**UST**	**NM_005715**	**-2.14**	**Uronyl-2-Sulfotransferase**
**HAGHL**	**NM_032304**	**-2.15**	**Hydroxyacylglutathione Hydrolase-Like**
**LOC202781**	**NR_028090**	**-2.15**	**PAXIP1 divergent transcript**
AIG1	NM_016108	-2.15	Androgen-Induced 1
KITLG	NM_000899	-2.15	KIT Ligand
STMN3	NM_015894	-2.15	Stathmin-Like 3
**MT3**	**NM_005954**	**-2.16**	**Metallothionein 3**
**NAGLU**	**NM_000263**	**-2.16**	**N-Acetylglucosaminidase Alpha**
**DENND2D**	**NM_024901**	**-2.17**	**DENN MADD Domain Containing 2D**
**FAM196B**	**NM_001129891**	**-2.17**	**Family With Sequence Similarity 196 Member B**
**LRP5**	**NM_002335**	**-2.17**	**Low Density Lipoprotein Receptor-Related Protein 5**
**MARCH9**	**NM_138396**	**-2.17**	**Membrane-Associated Ring Finger C3HC4 9**
**NLRC5**	**NM_032206**	**-2.17**	**NLR Family Card Domain Containing 5**
**REEP2**	**NM_016606**	**-2.17**	**Receptor Accessory Protein 2**
AKR1C2	NM_205845	-2.17	Aldo-Keto Reductase Family 1 Member C2
SVIL	NM_021738	-2.17	Supervillin
**APOBEC3B**	**NM_004900**	**-2.18**	**Apolipoprotein B mRNA Editing Enzyme Catalytic Polypeptide-Like 3B**
**TM7SF2**	**NM_003273**	**-2.18**	**Transmembrane 7 Superfamily Member 2**
LIMCH1	NM_014988	-2.18	LIM And Calponin Homology Domains 1
**ID1**	**NM_002165**	**-2.19**	**Inhibitor Of Dna Binding 1 Dominant Negative Helix-Loop-Helix Protein**
RTN1	NM_021136	-2.19	Reticulon 1
**PCYT2**	**NM_001184917**	**-2.2**	**Phosphate Cytidylyltransferase 2 Ethanolamine**
**RDH5**	**NM_001199771**	**-2.2**	**Retinol Dehydrogenase 5 11-Cis 9-Cis**
**APOL3**	**NM_145640**	**-2.21**	**Apolipoprotein L 3**

ENO2	NM_001975	-2.21	Enolase 2 Gamma Neuronal
**GFRA1**	**NM_005264**	**-2.22**	**GDNF Family Receptor Alpha 1**
**SNPH**	**NM_014723**	**-2.22**	**Syntaphilin**
GALNT5	NM_014568	-2.22	UDP-N-Acetyl-Alpha-D-Galactosamine Polypeptide N-Acetylgalactosaminyltransferase 5 Galnac-T5
B3GALNT1	NM_033169	-2.23	Beta-1 3-N-Acetylgalactosaminyltransferase 1 Globoside Blood Group
**TEC**	**NM_003215**	**-2.24**	**Tec Protein Tyrosine Kinase**
CPT1A	NM_001876	-2.24	Carnitine Palmitoyltransferase 1A Liver
MITF	NM_198159	-2.24	Microphthalmia-Associated Transcription Factor
ACSL5	NM_016234	-2.25	Acyl-Coa Synthetase Long-Chain Family Member 5
MX1	NM_001178046	-2.25	Myxovirus Influenza Virus Resistance 1 Interferon-Inducible Protein P78 Mouse
SPOCK3	NM_016950	-2.25	Sparc Osteonectin Cwcv And Kazal-Like Domains Proteoglycan Testican 3
STAMBPL1	NM_020799	-2.25	STAM Binding Protein-Like 1
DAPK1	NM_004938	-2.26	Death-Associated Protein Kinase 1
DBP	NM_001352	-2.26	D Site Of Albumin Promoter Albumin D-Box Binding Protein
CUEDC1	NM_017949	-2.27	CUE Domain Containing 1
EYA4	NM_172105	-2.27	Eyes Absent Homolog 4 Drosophila
**RALGDS**	**NM_006266**	**-2.28**	**Ral Guanine Nucleotide Dissociation Stimulator**
KHK	NM_006488	-2.28	Ketohexokinase Fructokinase
RND3	NM_005168	-2.28	Rho Family GTPase 3
ZFYVE28	NM_020972	-2.28	Zinc Finger FYVE Domain Containing 28
**MPZ**	**NM_000530**	**-2.29**	**Myelin Protein Zero**
AR	NM_000044	-2.29	Androgen Receptor
**SEMA4D**	**NM_006378**	**-2.3**	**Sema Domain Immunoglobulin Domain Ig Transmembrane Domain Tm And Short Cytoplasmic Domain Semaphorin 4D**
LYPD3	NM_014400	-2.3	LY6 PLAUR Domain Containing 3
ZNF385D	NM_024697	-2.31	Zinc Finger Protein 385D
ITGA7	NM_001144996	-2.32	Integrin Alpha 7
SMAD3	NM_005902	-2.32	SMAD Family Member 3
TMEM180	NM_024789	-2.32	Transmembrane Protein 180
**LETM2**	**NM_001199659**	**-2.33**	**Leucine Zipper-Ef-Hand Containing Transmembrane Protein 2**
**LOC344595**	**NR_028301**	**-2.33**	**DPPA2 upstream binding RNA**
ELF3	NM_004433	-2.33	E74-Like Factor 3 Ets Domain Transcription Factor Epithelial-Specific
IGFBP6	NM_002178	-2.33	Insulin-Like Growth Factor Binding Protein 6
NRP1	NM_003873	-2.33	Neuropilin 1
FAM115C	NM_001130025	-2.34	Family With Sequence Similarity 115 Member C
SERPINB9	NM_004155	-2.34	Serpin Peptidase Inhibitor Clade B Ovalbumin Member 9
**LOC729013**	**NR_034137**	**-2.35**	**ZBED5 antisense RNA 1**

ENPP2	NM_006209	-2.35	Ectonucleotide Pyrophosphatase Phosphodiesterase 2
RARRES1	NM_206963	-2.35	Retinoic Acid Receptor Responder Tazarotene Induced 1
**NME3**	**NM_002513**	**-2.36**	**NME NM23 Nucleoside Diphosphate Kinase 3**
MALL	NM_005434	-2.36	Mal T-Cell Differentiation Protein-Like
RDM1	NM_145654	-2.36	RAD52 Motif 1
**SLC16A4**	**NM_004696**	**-2.38**	**Solute Carrier Family 16 Member 4**
**CTSH**	**NM_004390**	**-2.39**	**Cathepsin H**
**NINJ1**	**NM_004148**	**-2.39**	**Ninjurin 1**
CDC42EP3	NM_006449	-2.39	CDC42 Effector Protein Rho GTPase Binding 3
FUCA1	NM_000147	-2.39	Fucosidase Alpha-L- 1 Tissue
PPL	NM_002705	-2.39	Periplakin
**CD38**	**NM_001775**	**-2.4**	**CD38 Molecule**
**LRRK2**	**NM_198578**	**-2.4**	**Leucine-Rich Repeat Kinase 2**
LPIN2	NM_014646	-2.4	Lipin 2
SLC4A4	NM_001134742	-2.4	Solute Carrier Family 4 Sodium Bicarbonate Cotransporter Member 4
**RGCC**	**NM_014059**	**-2.41**	**Regulator Of Cell Cycle**
**DENND3**	**NM_014957**	**-2.42**	**DENN MADD Domain Containing 2D Domain Containing 3**
**SEMA6B**	**NM_032108**	**-2.42**	**Sema Domain Transmembrane Domain TM And Cytoplasmic Domain Semaphorin 6B**
COL12A1	NM_004370	-2.43	Collagen Type XII Alpha 1
SSBP2	NM_012446	-2.43	Single-Stranded DNA Binding Protein 2
PAMR1	NM_001001991	-2.44	Peptidase Domain Containing Associated With Muscle Regeneration 1
RAPGEF1	NM_198679	-2.44	Rap Guanine Nucleotide Exchange Factor GEF 1
TM4SF1	NM_014220	-2.44	Transmembrane 4 L Six Family Member 1
PLEKHA6	NM_014935	-2.45	Pleckstrin Homology Domain Containing Family A Member 6
CXCL5	NM_002994	-2.46	Chemokine C-X-C Motif Ligand 5
**PARD6B**	**NM_032521**	**-2.48**	**Par-6 Family Cell Polarity Regulator Beta**
**PTPN22**	**NM_015967**	**-2.48**	**Protein Tyrosine Phosphatase Non-Receptor Type 22 Lymphoid**
RIN1	NM_004292	-2.48	Ras And Rab Interactor 1
**PSG3**	**NM_021016**	**-2.49**	**Pregnancy Specific Beta-1-Glycoprotein 3**
LMNA	NM_170707	-2.49	Lamin A C
BST2	NM_004335	-2.5	Bone Marrow Stromal Cell Antigen 2
**SDPR**	**NM_004657**	**-2.51**	**Serum Deprivation Response**
CTSO	NM_001334	-2.51	Cathepsin O
DPP4	NM_001935	-2.51	Dipeptidyl-Peptidase 4
GSE1	NM_014615	-2.53	Gse1 Coiled-Coil Protein
MET	NM_000245	-2.53	Met Proto-Oncogene
APOL1	NM_001136540	-2.54	Apolipoprotein L 1
**TPK1**	**NM_022445**	**-2.55**	**Thiamin Pyrophosphokinase 1**

COL8A1	NM_020351	-2.55	Collagen Type VIII Alpha 1
**SOD2**	**NM_001024465**	**-2.56**	**Superoxide Dismutase 2 Mitochondrial**
C3	NM_000064	-2.56	Complement Component 3
SLCO2B1	NM_007256	-2.57	Solute Carrier Organic Anion Transporter Family Member 2B1
**GPRC5C**	**NM_018653**	**-2.58**	**G Protein-Coupled Receptor Family C Group 5 Member C**
AKR1B10	NM_020299	-2.58	Aldo-Keto Reductase Family 1 Member B10 Aldose Reductase
**ARHGEF4**	**NM_015320**	**-2.59**	**Rho Guanine Nucleotide Exchange Factor GEF 4**
**PARP10**	**NM_032789**	**-2.59**	**Poly ADP-Ribose Polymerase Family Member 10**
AOX1	NM_001159	-2.59	Aldehyde Oxidase 1
CLDN3	NM_001306	-2.61	Claudin 3
MPP1	NM_002436	-2.61	Membrane Protein Palmitoylated 1 55KDa
PLEKHA2	NM_021623	-2.61	Pleckstrin Homology Domain Containing Family A Phosphoinositide Binding Specific Member 2
TMCO4	NM_181719	-2.61	Transmembrane And Coiled-Coil Domains 4
UBA7	NM_003335	-2.62	Ubiquitin-Like Modifier Activating Enzyme 7
**BHMT2**	**NM_017614**	**-2.66**	**Betaine--Homocysteine S-Methyltransferase 2**
ANXA1	NM_000700	-2.66	Annexin A1
DHRS13	NM_144683	-2.66	Dehydrogenase Reductase Sdr Family Member 13
FAM129A	NM_052966	-2.67	Family With Sequence Similarity 129 Member A
**DPYD**	**NM_000110**	**-2.68**	**Dihydropyrimidine Dehydrogenase**
**PLA2G16**	**NM_001128203**	**-2.68**	**Phospholipase A2 Group XVI**
PEG10	NM_001172438	-2.68	Paternally Expressed 10
CASP1	NM_033292	-2.69	Caspase 1 Apoptosis-Related Cysteine Peptidase
**HSD3B7**	**NM_025193**	**-2.71**	**Hydroxy-Delta-5-Steroid Dehydrogenase 3 Beta- And Steroid Delta-Isomerase 7**
BTC	NM_001729	-2.72	Betacellulin
**C6orf141**	**NM_001145652**	**-2.73**	**Chromosome 6 Open Reading Frame 141**
MANSC1	NM_018050	-2.74	MANSC Domain Containing 1
RARRES2	NM_002889	-2.75	Retinoic Acid Receptor Responder Tazarotene Induced 2
**SERPINB4**	**NM_002974**	**-2.76**	**Serpin Peptidase Inhibitor Clade B Ovalbumin Member 4**
MEST	NM_002402	-2.76	Mesoderm Specific Transcript
PDGFRA	NM_006206	-2.76	Platelet-Derived Growth Factor Receptor Alpha Polypeptide
C9orf89	NM_032310	-2.77	Chromosome 9 Open Reading Frame 89
ZDHHC23	NM_173570	-2.77	Zinc Finger DHHC-Type Containing 23
GPR39	NM_001508	-2.79	G Protein-Coupled Receptor 39
CD200	NM_001004196	-2.8	CD200 Molecule
KRT80	NM_182507	-2.8	Keratin 80
LOC643723	NR_038845	-2.81	LYPLAL1 divergent transcript
BNIP3	NM_004052	-2.82	BCL2 Adenovirus E1B 19Kda Interacting Protein 3
CFB	NM_001710	-2.82	Complement Factor B
RARRES3	NM_004585	-2.82	Retinoic Acid Receptor Responder Tazarotene Induced 3

OAS1	NM_016816	-2.83	2P-5P-Oligoadenylate Synthetase 1 40 46KDa
TMTC1	NM_001193451	-2.83	Transmembrane And Tetratricopeptide Repeat Containing 1
**SH3RF2**	**NM_152550**	**-2.84**	**SH3 Domain Containing Ring Finger 2**
ALDH3B1	NM_001030010	-2.85	Aldehyde Dehydrogenase 3 Family Member B1
MGST1	NM_020300	-2.86	Microsomal Glutathione S-Transferase 1
ATP2B4	NM_001684	-2.88	ATPase Ca Transporting Plasma Membrane 4
TMEM158	NM_015444	-2.88	Transmembrane Protein 158 Gene Pseudogene
KIAA1199	NM_018689	-2.89	KIAA1199
**KCNAB2**	**NM_001199862**	**-2.9**	**Potassium Voltage-Gated Channel Shaker-Related Subfamily Beta Member 2**
ARID5B	NM_032199	-2.9	AT Rich Interactive Domain 5B MRF1-Like
G0S2	NM_015714	-2.92	G0 G1Switch 2
ADORA2B	NM_000676	-2.93	Adenosine A2B Receptor
DIO2	NM_013989	-2.94	Deiodinase Iodothyronine Type Ii
MYO5C	NM_018728	-2.95	Myosin VC
OCLN	NM_002538	-2.95	Occludin
CXCL3	NM_002090	-2.96	Chemokine C-X-C Motif Ligand 3
TNFRSF6B	NM_003823	-2.96	Tumor Necrosis Factor Receptor Superfamily Member 6B Decoy
FAIM2	NM_012306	-2.97	Fas Apoptotic Inhibitory Molecule 2
COL17A1	NM_000494	-2.98	Collagen Type XVII Alpha 1
CSF1	NM_000757	-3	Colony Stimulating Factor 1 Macrophage
GALNT12	NM_024642	-3.01	UDP-N-Acetyl-Alpha-D-Galactosamine Polypeptide N-Acetylgalactosaminyltransferase 12 Galnac-T12
SGSH	NM_000199	-3.01	N-Sulfoglucosamine Sulfohydrolase
TSPAN18	NM_130783	-3.02	Tetraspanin 18
ANKRD29	NM_173505	-3.03	Ankyrin Repeat Domain 29
FRZB	NM_001463	-3.04	Frizzled-Related Protein
LPAR1	NM_001401	-3.04	Lysophosphatidic Acid Receptor 1
LURAP1L	NM_203403	-3.04	Leucine Rich Adaptor Protein 1-Like
METTL7B	NM_152637	-3.05	Methyltransferase Like 7B
LINC00341	NR_026779	-3.06	Long Intergenic Non-Protein Coding RNA 341
HHEX	NM_002729	-3.07	Hematopoietically Expressed Homeobox
RBM47	NM_001098634	-3.08	RNA Binding Motif Protein 47
FOSL1	NM_005438	-3.09	FOS-Like Antigen 1
ABCG2	NM_004827	-3.1	ATP-Binding Cassette Sub-Family G White Member 2
DHRS3	NM_004753	-3.15	Dehydrogenase Reductase Sdr Family Member 3
KCNN4	NM_002250	-3.16	Potassium Intermediate Small Conductance Calcium-Activated Channel Subfamily N Member 4
KLHL4	NM_019117	-3.16	Kelch-Like Family Member 4
CYP1B1	NM_000104	-3.18	Cytochrome P450 Family 1 Subfamily B Polypeptide 1
IFI35	NM_005533	-3.18	Interferon-Induced Protein 35

TCF21	NM_003206	-3.21	Transcription Factor 21
BEX1	NM_018476	-3.22	Brain Expressed X-Linked 1
CCDC85B	NM_006848	-3.24	Coiled-Coil Domain Containing 85B
VWA5A	NM_014622	-3.26	Von Willebrand Factor A Domain Containing 5A
BIRC3	NM_182962	-3.27	Baculoviral Iap Repeat Containing 3
SLC22A23	NM_015482	-3.27	Solute Carrier Family 22 Member 23
**PARD6A**	**NM_016948**	**-3.28**	**Par-6 Family Cell Polarity Regulator Alpha**
IL6R	NM_000565	-3.29	Interleukin 6 Receptor
HGF	NM_000601	-3.31	Hepatocyte Growth Factor Hepapoietin A Scatter Factor
THSD4	NM_024817	-3.33	Thrombospondin Type I Domain Containing 4
AREG	NM_001657	-3.34	Amphiregulin
KYNU	NM_003937	-3.4	Kynureninase
PNMA2	NM_007257	-3.4	Paraneoplastic Ma Antigen 2
SH3BP4	NM_014521	-3.42	SH3-Domain Binding Protein 4
COL4A6	NM_033641	-3.45	Collagen Type IV Alpha 6
AQP5	NM_001651	-3.5	Aquaporin 5
RAB27B	NM_004163	-3.51	RAB27B Member RAS Oncogene Family
CRABP2	NM_001878	-3.52	Cellular Retinoic Acid Binding Protein 2
GMPR	NM_006877	-3.53	Guanosine Monophosphate Reductase
SLC7A4	NM_004173	-3.54	Solute Carrier Family 7 Member 4
PDE7B	NM_018945	-3.55	Phosphodiesterase 7B
SLC48A1	NM_017842	-3.58	Solute Carrier Family 48 Heme Transporter Member 1
CNTN3	NM_020872	-3.63	Contactin 3 Plasmacytoma Associated
HPSE	NM_001098540	-3.72	Heparanase
TGFBR3	NM_003243	-3.72	Transforming Growth Factor Beta Receptor Iii
TOX2	NM_001098797	-3.72	TOX High Mobility Group Box Family Member 2
BMP4	NM_130851	-3.73	Bone Morphogenetic Protein 4
PPP1R9A	NM_001166161	-3.73	Protein Phosphatase 1 Regulatory Subunit 9A
SYNE3	NM_152592	-3.79	Spectrin Repeat Containing Nuclear Envelope Family Member 3
TSLP	NM_033035	-3.8	Thymic Stromal Lymphopoietin
MRGPRF	NM_145015	-3.81	MAS-Related GPR Member F
PLAT	NM_000930	-3.81	Plasminogen Activator Tissue
P2RX6	NM_005446	-3.82	Purinergic Receptor P2X Ligand-Gated Ion Channel 6
AKR1C1	NM_001353	-3.84	Aldo-Keto Reductase Family 1 Member C1
CARD16	NM_052889	-3.85	Caspase Recruitment Domain Family Member 16
NR2F2	NM_021005	-3.85	Nuclear Receptor Subfamily 2 Group F Member 2
ARHGAP29	NM_004815	-3.86	Rho GTPase Activating Protein 29
LAMA3	NM_198129	-3.87	Laminin Alpha 3
PBX1	NM_002585	-3.89	Pre-B-Cell Leukemia Homeobox 1
ANK1	NM_001142446	-3.93	Ankyrin 1 Erythrocytic

ADM	NM_001124	-3.94	Adrenomedullin
LYPD6B	NM_177964	-3.97	LY6 PLAUR Domain Containing 6B
METTL7A	NM_014033	-3.97	Methyltransferase Like 7A
BMPER	NM_133468	-3.99	BMP Binding Endothelial Regulator
LRRN3	NM_018334	-3.99	Leucine Rich Repeat Neuronal 3
PHLDA1	NM_007350	-4.05	Pleckstrin Homology-Like Domain Family A Member 1
ATOH8	NM_032827	-4.09	Atonal Homolog 8 Drosophila
NPTX1	NM_002522	-4.12	Neuronal Pentraxin I
IL18	NM_001562	-4.18	Interleukin 18 Interferon-Gamma-Inducing Factor
MYPN	NM_032578	-4.19	Myopalladin
NDP	NM_000266	-4.19	Norrie Disease Pseudoglioma
SEMA3D	NM_152754	-4.19	Sema Domain Immunoglobulin Domain Ig Short Basic Domain Secreted Semaphorin 3D
GABRB1	NM_000812	-4.26	Gamma-Aminobutyric Acid GABA A Receptor Beta 1
TM4SF18	NM_001184723	-4.29	Transmembrane 4 L Six Family Member 18
PLEKHG4	NM_001129728	-4.32	Pleckstrin Homology Domain Containing Family G With Rhogef Domain Member 4
C10orf54	NM_022153	-4.36	Chromosome 10 Open Reading Frame 54
GBP2	NM_004120	-4.39	Guanylate Binding Protein 2 Interferon-Inducible
IKZF2	NM_016260	-4.42	IKAROS Family Zinc Finger 2 Helios
MGARP	NM_032623	-4.42	Mitochondria-Localized Glutamic Acid-Rich Protein
CNTNAP1	NM_003632	-4.49	Contactin Associated Protein 1
HPCAL1	NM_002149	-4.5	Hippocalcin-Like 1
CDH6	NM_004932	-4.56	Cadherin 6 Type 2 K-Cadherin Fetal Kidney
CXADR	NM_001338	-4.56	Coxsackie Virus And Adenovirus Receptor
PPAP2B	NM_003713	-4.7	Phosphatidic Acid Phosphatase Type 2B
**FAM20C**	**NM_020223**	**-4.77**	**Family With Sequence Similarity 20 Member C**
F3	NM_001993	-4.78	Coagulation Factor Iii Thromboplastin Tissue Factor
PTX3	NM_002852	-4.85	Pentraxin 3 Long
CA12	NM_001218	-4.97	Carbonic Anhydrase Xii
**C1QTNF1**	**NM_030968**	**-5.01**	**C1q And Tumor Necrosis Factor Related Protein 1**
DIRAS3	NM_004675	-5.03	DIRAS Family Gtp-Binding Ras-Like 3
ZNF395	NM_018660	-5.56	Zinc Finger Protein 395
CLDN11	NM_005602	-5.63	Claudin 11
CXCL2	NM_002089	-5.69	Chemokine C-X-C Motif Ligand 2
KRT19	NM_002276	-5.73	Keratin 19
EVI2B	NM_006495	-5.86	Ecotropic Viral Integration Site 2B
GPRC5A	NM_003979	-5.87	G Protein-Coupled Receptor Family C Group 5 Member A
GDF5	NM_000557	-5.97	Growth Differentiation Factor 5
COL13A1	NM_001130103	-6.24	Collagen Type XIII Alpha 1
FAM65C	NM_080829	-6.42	Family With Sequence Similarity 65 Member C

TNFRSF1B	NM_001066	-6.42	Tumor Necrosis Factor Receptor Superfamily Member 1B
SFRP1	NM_003012	-6.53	Secreted Frizzled-Related Protein 1
SECTM1	NM_003004	-6.55	Secreted And Transmembrane 1
COLEC12	NM_130386	-7.18	Collectin Sub-Family Member 12
VCAM1	NM_001078	-7.31	Vascular Cell Adhesion Molecule 1
AGPAT9	NM_032717	-7.52	1-Acylglycerol-3-Phosphate O-Acyltransferase 9
SERPINB2	NM_002575	-7.61	Serpin Peptidase Inhibitor Clade B Ovalbumin Member 2
ANXA10	NM_007193	-7.66	Annexin A10
PTGES	NM_004878	-8.06	Prostaglandin E Synthase
CPA4	NM_016352	-11.87	Carboxypeptidase A4

* Fold Change relative to Control

Bolded genes are unique DEGs for TGF-β2
