# Supplementary material for: TGF-β Promotes Endothelial-to-Mesenchymal Transition and Alters Corneal Endothelial Cell Migration in Fuchs Endothelial Corneal Dystrophy
Source: Int J Mol Sci. 2025 Jul 11;26(14):6685. doi: 10.3390/ijms26146685 (PMC12294433; doi:10.3390/ijms26146685)
Supplement: Supplementary file 1 [file ijms-26-06685-s001.zip › Table S3 EMT Gene List.pdf]

Table S3. Gene set that contributed to the enrichment of the Epithelial to Mesenchymal pathway in FECD-SVF5-54F with TGF-β1 or TGF-β2

Gene Symbol	RefSeq	FC for TGF-β1*	FC for TGF-β2*	Gene Name
ABI3BP	NM_015429	-1.79	-2.05	ABI Family Member 3 NESH Binding Protein
ACTA2	NM_001613	2.48	2.73	Actin Alpha 2 Smooth Muscle Aorta
ADAM12	NM_003474	2.74	2.76	ADAM Metallopeptidase Domain 12
APLP1	NM_001024807	1.16	1.04	Amyloid Beta A4 Precursor-Like Protein 1
AREG	NM_001657	-3.25	-3.34	Amphiregulin
BASP1	NM_006317	-1.05	1.02	Brain Abundant Membrane Attached Signal Protein 1
BDNF	NM_001143810	-1	-1	Brain-Derived Neurotrophic Factor
BMP1	NM_006129	2.41	2.37	Bone Morphogenetic Protein 1
CADM1	NM_014333	1.25	-1.03	Cell Adhesion Molecule 1
CALD1	NM_033138	1.51	1.7	Caldesmon 1
CALU	NM_001219	1.2	1.13	Calumenin
CD44	NM_000610	-1.44	-1.45	CD44 Molecule Indian Blood Group
CD59	NM_001127223	1.32	1.62	CD59 Molecule Complement Regulatory Protein
CDH11	NM_001797	2.26	2.54	Cadherin 11 Type 2 OB-Cadherin Osteoblast
CDH2	NM_001792	1.78	1.81	Cadherin 2 Type 1 N-Cadherin Neuronal
CDH6	NM_004932	-4.27	-4.56	Cadherin 6 Type 2 K-Cadherin Fetal Kidney
COL12A1	NM_004370	-2.22	-2.43	Collagen Type XII Alpha 1
COL16A1	NM_001856	1.64	1.79	Collagen Type XVI Alpha 1
COL1A1	NM_000088	2.84	3.13	Collagen Type I Alpha 1
COL1A2	NM_000089	2.09	2.28	Collagen Type I Alpha 2
COL3A1	NM_000090	1.15	1.08	Collagen Type III Alpha 1
COL4A1	NM_001845	3.63	3.52	Collagen Type IV Alpha 1
COL4A2	NM_001846	2.78	2.78	Collagen Type IV Alpha 2
COL5A2	NM_000393	-1.25	-1.13	Collagen Type V Alpha 2
COL5A3	NM_015719	3.24	3.59	Collagen Type V Alpha 3
COL8A2	NM_005202	1.11	1.19	Collagen Type VIII Alpha 2
COMP	NM_000095	1.77	1.72	Cartilage Oligomeric Matrix Protein
COPA	NM_001098398	1.01	1.17	Coatomer Protein Complex Subunit Alpha
CRLF1	NM_004750	2.38	3.57	Cytokine Receptor-Like Factor 1
CTGF	NM_001901	6.12	6.81	Connective Tissue Growth Factor
CTHRC1	NM_138455	3.6	4.24	Collagen Triple Helix Repeat Containing 1
CXCL1	NM_001511	-1.75	-2.12	Chemokine C-X-C Motif Ligand 1 Melanoma Growth Stimulating Activity Alpha
CXCL12	NM_001178134	1.75	1.76	Chemokine C-X-C Motif Ligand 12
CXCL6	NM_002993	-1.57	-2.09	Chemokine C-X-C Motif Ligand 6
CYR61	NM_001554	1.07	1.07	Cysteine-Rich Angiogenic Inducer 61
DCN	NM_133503	-1.08	-1.2	Decorin
DKK1	NM_012242	-1.88	-2.22	Dickkopf WNT Signaling Pathway Inhibitor 1
DPYSL3	NM_001197294	1.1	-1.17	Dihydropyrimidinase-Like 3
DST	NM_015548	-1.95	-1.89	Dystonin
ECM2	NM_001393	-1	-1	Extracellular Matrix Protein 2 Female Organ And Adipocyte Specific
EDIL3	NM_005711	-1.07	-1.25	EGF-Like Repeats And Discoidin I-Like Domains 3
EMP3	NM_001425	-1.73	-1.68	Epithelial Membrane Protein 3
ENO2	NM_001975	-2	-2.21	Enolase 2 Gamma Neuronal
FAP	NM_004460	4.35	4.07	Fibroblast Activation Protein Alpha
FAS	NM_000043	-1.98	-1.52	Fas Cell Surface Death Receptor
FBN1	NM_000138	1.41	1.52	Fibrillin 1
FBN2	NM_001999	1.25	1.13	Fibrillin 2
FERMT2	NM_001134999	1.21	1.29	Fermitin Family Member 2

FGF2	NM_002006	-1.14	-1.24	Fibroblast Growth Factor 2 Basic
FLNA	NM_001110556	1.1	1.16	Filamin A Alpha
FOXC2	NM_005251	4.95	4.52	Forkhead Box C2 MFH-1 Mesenchyme Forkhead 1
FSTL1	NM_007085	1.49	1.43	Follistatin-Like 1
FSTL3	NM_005860	8.51	8.82	Follistatin-Like 3 Secreted Glycoprotein
FUCA1	NM_000147	-2.45	-2.39	Fucosidase Alpha-L- 1 Tissue
FZD8	NM_031866	1.61	1.77	Frizzled Family Receptor 8
GADD45A	NM_001924	-1.05	-1.22	Growth Arrest And DNA-Damage-Inducible Alpha
GADD45B	NM_015675	4.88	4.58	Growth Arrest And DNA-Damage-Inducible Beta
GAS1	NM_002048	-1.11	-1.03	Growth Arrest-Specific 1
GEM	NM_005261	-1.08	1.06	GTP Binding Protein Overexpressed In Skeletal Muscle
GLIPR1	NM_006851	-1.07	-1.08	GLI Pathogenesis-Related 1
GPX7	NM_015696	1.12	-1.05	Glutathione Peroxidase 7
GREM1	NM_013372	3.15	3.49	Gremlin 1 DAN Family Bmp Antagonist
HTRA1	NM_002775	-1.33	-1.25	HtrA Serine Peptidase 1
ID2	NM_002166	-3.96	-3.95	Inhibitor Of DNA Binding 2 Dominant Negative Helix-Loop-Helix Protein
IGFBP2	NM_000597	1.07	-1.01	Insulin-Like Growth Factor Binding Protein 2 36Kda
IGFBP4	NM_001552	1	-1.02	Insulin-Like Growth Factor Binding Protein 4
IL15	NM_000585	1.13	-1.37	Interleukin 15
IL32	NM_001012718	2.37	2.72	Interleukin 32
IL6	NM_000600	1.94	1.67	Interleukin 6 Interferon Beta 2
INHBA	NM_002192	4.77	4.76	Inhibin Beta A
ITGA2	NM_002203	1.11	-1.09	Integrin Alpha 2 CD49B Alpha 2 Subunit Of VLA-2 Receptor
ITGAV	NM_002210	2.21	2.15	Integrin Alpha V
ITGB1	NM_002211	1.29	1.45	Integrin Beta 1 Fibronectin Receptor Beta Polypeptide Antigen CD29 Includes MDF2 MSK12
ITGB3	NM_000212	1.7	1.36	Integrin Beta 3 Platelet Glycoprotein Iiia Antigen Cd61
ITGB5	NM_002213	1.21	-1.02	Integrin Beta 5
JUN	NM_002228	-1.15	1.2	Jun Proto-Oncogene
LAMA1	NM_005559	-1.58	-1.55	Laminin Alpha 1
LAMA3	NM_198129	-3	-3.87	Laminin Alpha 3
LAMC1	NM_002293	1.36	1.27	Laminin Gamma 1 Formerly LAMB2
LAMC2	NM_005562	10.11	10.63	Laminin Gamma 2
LOX	NM_002317	1.28	1.3	Lysyl Oxidase
LOXL1	NM_005576	1.14	1.12	Lysyl Oxidase-Like 1
LOXL2	NM_002318	-1.36	-1.46	Lysyl Oxidase-Like 2
MAGEE1	NM_020932	1.06	1.25	Melanoma Antigen Family E 1
MATN3	NM_002381	2.45	2.73	Matrilin 3
MCM7	NM_005916	-1.12	-1.05	Minichromosome Maintenance Complex Component 7
MFAP5	NM_003480	-1	1.33	Microfibrillar Associated Protein 5
MGP	NM_001190839	-1	-1	Matrix Gla Protein
MMP14	NM_004995	1.87	1.61	Matrix Metallopeptidase 14 Membrane-Inserted
MMP2	NM_004530	3.57	4.11	Matrix Metallopeptidase 2 Gelatinase A 72KDa Gelatinase 72KDa Type Iv Collagenase
MMP3	NM_002422	1.52	-1.27	Matrix Metallopeptidase 3 Stromelysin 1 Progelatinase
MSX1	NM_002448	1.01	1	Msh Homeobox 1
MXRA5	NM_015419	-1.09	1.36	Matrix-Remodelling Associated 5
MYLK	NM_053025	-2.16	-2.23	Myosin Light Chain Kinase

NID2	NM_007361	-1.51	-1.44	Nidogen 2 Osteonidogen
NNMT	NM_006169	1.1	1.21	Nicotinamide N-Methyltransferase
NT5E	NM_002526	-1.18	-1.09	5P-Nucleotidase Ecto CD73
NTM	NM_001144058	4.02	4.06	Neurotrimin
OXTR	NM_000916	-1.15	-1.14	Oxytocin Receptor
PCOLCE	NM_002593	-1.17	1.11	Procollagen C-Endopeptidase Enhancer
PCOLCE2	NM_013363	-1.07	1.1	Procollagen C-Endopeptidase Enhancer 2
PDLIM4	NM_003687	1.68	1.6	PDZ And LIM Domain 4
PFN2	NM_053024	-1.67	-1.63	Profilin 2
PLAUR	NM_002659	1.07	-1.01	Plasminogen Activator Urokinase Receptor
PLOD1	NM_000302	1.15	1.17	Procollagen-Lysine 2-Oxoglutarate 5-Dioxygenase 1
PLOD2	NM_182943	1.04	-1.22	Procollagen-Lysine 2-Oxoglutarate 5-Dioxygenase 2
PMP22	NM_153322	-1.32	-1.3	Peripheral Myelin Protein 22
POSTN	NM_006475	2.33	2.01	Periostin Osteoblast Specific Factor
PPIB	NM_000942	1.28	1.32	Peptidylprolyl Isomerase B Cyclophilin B
PRRX1	NM_022716	1.2	1.5	Paired Related Homeobox 1
PTHLH	NM_198965	6.88	7.54	Parathyroid Hormone-Like Hormone
PTX3	NM_002852	-4.6	-4.85	Pentraxin 3 Long
RGS4	NM_001102445	-1.93	-2.14	Regulator Of G-Protein Signaling 4
RHOB	NM_004040	1.74	2.02	Ras Homolog Family Member B
SCG2	NM_003469	-1.5	-1.31	Secretogranin Ii
SDC1	NM_002997	1.62	1.67	Syndecan 1
SDC4	NM_002999	-1.57	-1.64	Syndecan 4
SERPINE1	NM_000602	3.3	3.7	Serpin Peptidase Inhibitor Clade E Nexin Plasminogen Activator Inhibitor Type 1 Member 1
SERPINE2	NM_006216	3.85	3.84	Serpin Peptidase Inhibitor Clade E Nexin Plasminogen Activator Inhibitor Type 1 Member 2
SFRP1	NM_003012	-4.44	-6.53	Secreted Frizzled-Related Protein 1
SFRP4	NM_003014	-1	-1	Secreted Frizzled-Related Protein 4
SGCB	NM_000232	-1.06	-1.21	Sarcoglycan Beta 43KDa Dystrophin-Associated Glycoprotein
SGCD	NM_001128209	-1	-1	Sarcoglycan Delta 35KDa Dystrophin-Associated Glycoprotein
SGCG	NM_000231	1.22	1.15	Sarcoglycan Gamma 35KDa Dystrophin-Associated Glycoprotein
SLC6A8	NM_005629	1.12	1.04	Solute Carrier Family 6 Neurotransmitter Transporter Member 8
SLIT2	NM_004787	-1.47	-1.71	Slit Homolog 2 Drosophila
SLIT3	NM_003062	1.83	1.5	Slit Homolog 3 Drosophila
SNAI2	NM_003068	1.63	2.07	Snail Family Zinc Finger 2
SNTB1	NM_021021	-1.14	-1.45	Syntrophin Beta 1 Dystrophin-Associated Protein A1 59KDa Basic Component 1
SPARC	NM_003118	2.03	2.03	Secreted Protein Acidic Cysteine-Rich Osteonectin
SPOCK1	NM_004598	4.71	4.35	Sparc Osteonectin Cwcv And Kazal-Like Domains Proteoglycan Testican 1
SPP1	NM_001040058	2.45	1.63	Secreted Phosphoprotein 1
TFPI2	NM_006528	6.4	7.88	Tissue Factor Pathway Inhibitor 2
TGFB1	NM_000660	2.53	2.65	Transforming Growth Factor Beta 1
TGFBI	NM_000358	6.61	7.13	Transforming Growth Factor Beta-Induced 68KDa
TGFBR3	NM_003243	-4.95	-3.72	Transforming Growth Factor Beta Receptor Iii
THBS1	NM_003246	4.76	4.89	Thrombospondin 1
THBS2	NM_003247	-1.04	-1	Thrombospondin 2
THY1	NM_006288	1.11	1.15	Thy-1 Cell Surface Antigen
TIMP1	NM_003254	1.18	1.55	Timp Metallopeptidase Inhibitor 1
TIMP3	NM_000362	1.17	1.42	Timp Metallopeptidase Inhibitor 3

TNFAIP3	NM_006290	-1.84	-2.17	Tumor Necrosis Factor Alpha-Induced Protein 3
TNFRSF11B	NM_002546	-1.12	1.02	Tumor Necrosis Factor Receptor Superfamily Member 11B
TNFRSF12A	NM_016639	1.61	1.61	Tumor Necrosis Factor Receptor Superfamily Member 12A
VCAM1	NM_001078	-4.97	-7.31	Vascular Cell Adhesion Molecule 1
VCAN	NM_004385	5.6	4.12	Versican
VEGFA	NM_001171623	2.24	2.06	Vascular Endothelial Growth Factor A
VEGFC	NM_005429	1.09	1.01	Vascular Endothelial Growth Factor C
WIPF1	NM_003387	-1.09	-1.04	WAS WASL Interacting Protein Family Member 1

* Fold Change relative to Control
